# Dissecting contributions of pulmonary arterial remodeling to right ventricular afterload in pulmonary hypertension

**DOI:** 10.1002/btm2.70035

**Published:** 2025-06-26

**Authors:** Sunder Neelakantan, Emilio A. Mendiola, Byron Zambrano, Alexander Vang, Kyle J. Myers, Peng Zhang, Gaurav Choudhary, Reza Avazmohammadi

**Affiliations:** ^1^ Department of Biomedical Engineering Texas A&M University College Station Texas USA; ^2^ J. Mike Walker’66 Department of Mechanical Engineering Texas A&M University College Station Texas USA; ^3^ Warren Alpert Medical School of Brown University Providence Rhode Island USA; ^4^ Hagler Institute of Advanced Study, Texas A&M University College Station Texas USA; ^5^ Department of Cardiovascular Sciences Houston Methodist Academic Institute Houston Texas USA

**Keywords:** Pulmonary hypertension, pulmonary heomdynamics, 1D fluid structure interaction, characteristic impedance

## Abstract

Pulmonary hypertension (PH) is defined as an elevation in the right ventricular (RV) afterload, characterized by increased hemodynamic pressure in the main pulmonary artery (PA). Elevations in RV afterload increase RV wall stress, resulting in RV remodeling and potentially RV failure. From a biomechanical standpoint, the primary drivers for RV afterload elevations include increases in pulmonary vascular resistance (PVR) in the distal vasculature and decreases in vessel compliance in the proximal arteries. However, the individual contributions of the various vascular remodeling events toward the progression of PA pressure elevations and altered vascular hemodynamics remain elusive. In this study, we used a subject‐specific one‐dimensional (1D) fluid–structure interaction (FSI) model to investigate the alteration of pulmonary hemodynamics in PH and to quantify the contributions of decreased compliance and increased resistance toward increased main pulmonary artery (MPA) pressure. We used a combination of subject‐specific hemodynamic measurements, ex‐vivo mechanical testing and histological analysis of arterial tissue specimens, and ex‐vivo x‐ray micro‐tomography imaging to develop the 1D FSI model and dissect the contribution of PA remodeling events toward alterations in the MPA pressure waveform. Both the amplitude and pulsatility of the MPA pressure waveform were analyzed. Our results indicated that increased distal resistance has the greatest effect on the increase in maximum MPA pressure, while decreased vessel compliance caused significant elevations in the characteristic impedance. The method presented in this study will serve as an essential step toward understanding the complex interplay between PA remodeling events that lead to the most adverse effect on RV function.

**Translational Impact Statement**

Pulmonary hypertension (PH) causes an elevation in pulmonary arterial pressure and is associated with a mortality rate of 4.5–12.3 deaths per 100,000 individuals per year. Right ventricular (RV) remodeling and failure, triggered by this pressure overload, is the primary cause of death. However, the interaction between remodeling events at the arterial side, causing the overload, and the remodeling events at the RV side remains poorly understood. This study offers a rigorous tool to quantify the individual contribution of each vascular remodeling event toward the RV afterload, thus enabling computational phenotyping of PH and improving individualized treatment based on quantified contributions.

## 1 INTRODUCTION

Pulmonary hypertension (PH) is characterized by an elevation in mean pulmonary arterial pressure (mPAP) and affects ~1% of the world population.^1^ PH can be subdivided into several etiologies, with group 1 PH being known as pulmonary arterial hypertension (PAH). The increase in mPAP in PAH and the subsequent increase in the right ventricular (RV) afterload is due to an increase in pulmonary vascular resistance (PVR) and a decrease in pulmonary arterial compliance (PAC).^1^, ^2^, ^3^, ^4^ In turn, the elevations in RV afterload lead to increased RV wall stress,^5^, ^6^ triggering a cascade of mechano‐driven RV remodeling events.^7^, ^8^, ^9^, ^10^, ^11^ Left untreated, RV remodeling can lead to right heart failure and death. The standardized death rate in PH was reported to be 4.5 to 12.3 in a 100,000 population^12^ and the median survival was reported to be 7 years for patients with PAH and 2.8 years for patients with idiopathic PAH.^13^, ^14^, ^15^ As RV remodeling is primarily triggered by increases in RV afterload, understanding the individual contribution of each vascular remodeling event toward the elevations of RV afterload is essential. Also, as each pulmonary arterial (PA) remodeling event may require different clinical intervention strategies, there is a need to develop tools that can quantify the effects of isolated remodeling events on RV afterload and PA pressure in a patient‐specific manner to enable optimized treatments. The development of such tools remains an unmet need in PH.

The primary tissue‐level remodeling events in the pulmonary vasculature driving increased PA pressure and RV afterload are increased resistance and decreased vessel compliance (increased vessel stiffness). Increased resistance is predominantly caused by the narrowing of the lumen in the distal vessels, including precapillary arterioles,^16^, ^17^ and the “pruning” of distal arteries.^1^, ^2^ Increased vessel stiffness occurs through smooth muscle hypertrophy and proliferation of vascular cells, which contribute to increased deposition of extracellular matrix components. However, distinguishing the contributions of increased resistance and decreased compliance to afterload remains difficult due to the inadequacy of existing in‐vivo indices that aim to assess these contributions. In contrast, in‐vivo measurements, such as catheterization and medical imaging, combined with in‐silico modeling,^18^, ^19^, ^20^ offer an innovative approach to investigate patient‐specific pulmonary hemodynamics and quantify the contribution of increased stiffness and resistance.

Several studies^21^, ^22^, ^23^, ^24^, ^25^, ^26^ have significantly advanced the use of in‐silico modeling approaches to investigate pulmonary hemodynamics in the past decade. Such studies have developed three‐dimensional (3D) computational fluid‐structure interaction (FSI) models of the proximal vasculature to analyze hemodynamic behavior in various vascular diseases, including PH. In such studies, the structure of the proximal vasculature is obtained through medical imaging, and the distal vasculature is represented by Windkessel elements. The flow boundary conditions used in such studies are generally obtained through catheterization and/or 4D flow magnetic resonance imaging (MRI). These models^21^, ^22^, ^23^, ^24^, ^25^, ^26^ allow for high‐fidelity simulations and the incorporation of subject‐specific vascular geometry, enabling the accurate assessment of parameters such as wall shear stress (WSS) and vorticity. In addition, high‐fidelity models have also been used for phenotyping studies in the systemic circulation.^27^, ^28^ Such studies reconstructed the geometry of the aorta from rodent images and used 3D FSI to investigate hemodynamics in systemic hypertension. High‐fidelity FSI models become especially important when investigating systemic circulation due to the complex geometry of the aorta. Due to their higher fidelity, 3D FSI models also tend to be better suited to analyze turbulent flow behavior. While such models enable the in‐silico simulation of physiologically realistic flow in the proximal vessels, they can be computationally expensive when incorporating the actual geometry of the distal vasculature. This limits the potential application of high‐fidelity 3D FSI models in clinical settings.

In contrast, other studies have developed reduced‐order modeling approaches to reduce computational time and cost while including the distal vasculature. One such approach that has recently gained interest, especially in terms of subject‐specific simulations, is the 1D FSI model.^29^, ^30^, ^31^, ^32^, ^33^, ^34^ These models tend to approximate vessels as a line with a given length and lumen area and work under the assumption of laminar and axisymmetric flow. Studies have demonstrated the ability of 1D FSI models of the vascular system to capture physiologically realistic pressures in the vessels.^29^, ^31^ Such studies have also reported the capacity to include a significant portion of the pulmonary vasculature (up to 400 vessels), with the number of vessels being limited by the imaging modality. A limitation of 1D FSI models is their inability to describe small‐scale flow characteristics, such as vorticity. However, studies investigating pulmonary blood flow have reported minimal turbulence in the distal vasculature.^21^, ^24^ Thus, the reduction in fidelity in 1D FSI models leads to minimal loss of accuracy when investigating the alteration in pressure and flow pulsatility in the pulmonary vasculature due to PH. Such models with reduced fidelity significantly reduce computational costs while offering an accurate estimation of pressure and vessel stress, facilitating the translation of such models into clinical applications. However, reduced‐order models have yet to be used to address the need for an improved understanding of the contributions of individual PA remodeling events toward altered vascular hemodynamics in PH.

**FIGURE 1:**
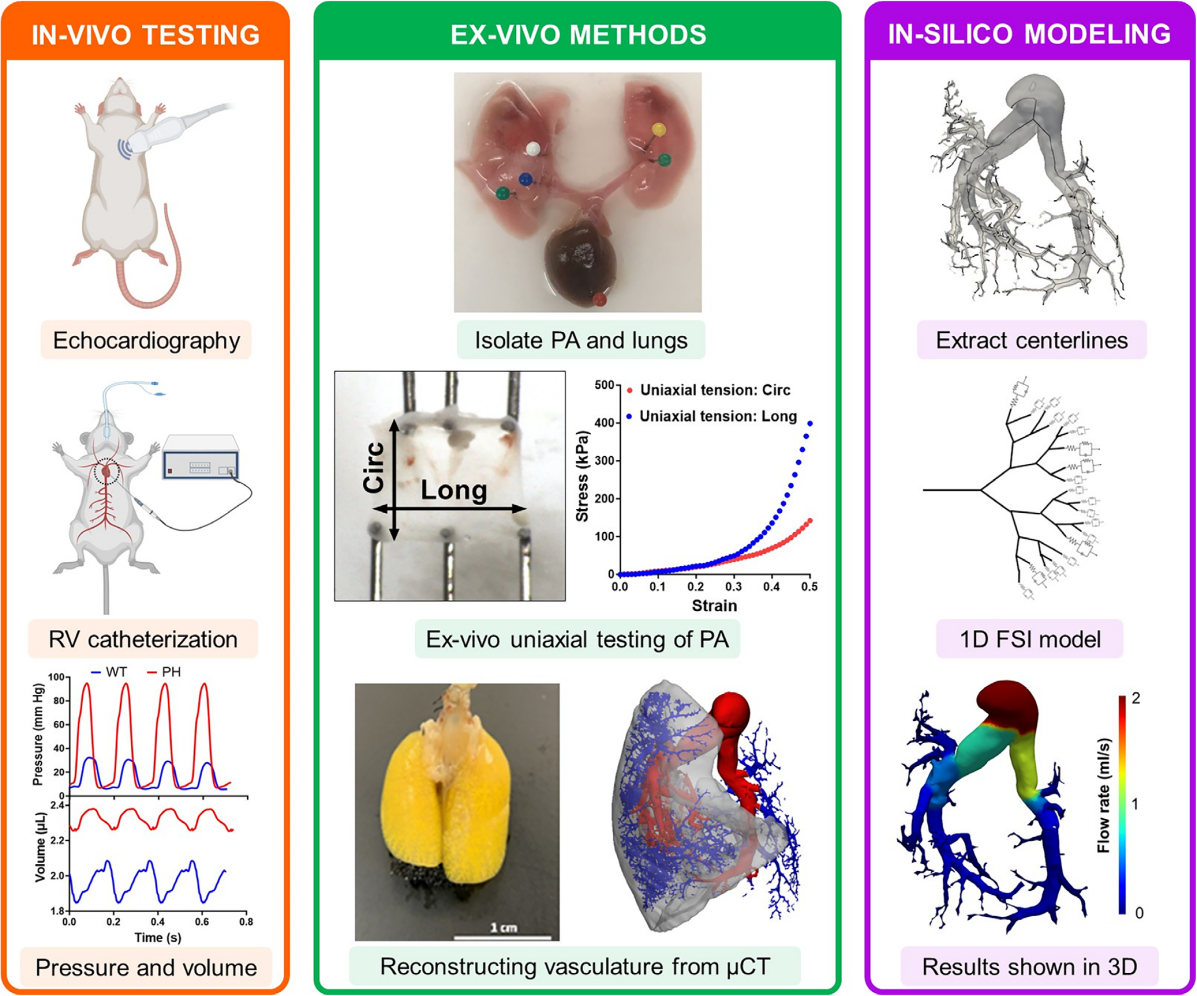
Combined in‐vivo, ex‐vivo, and in‐silico approaches were used to separate the contribution of individual remodeling events toward increased MPA pressure. Data from in‐vivo and ex‐vivo measurements were combined to create an in‐silico model of the pulmonary vasculature. Circ, circumferential; CTL, control; FSI, fluid structure interaction; Long, longitudinal; MPA, main pulmonary artery; PA, pulmonary artery; PH, pulmonary hypertension; μCT, x‐ray microtomography.

In this study, a recently developed 1D FSI model^29^, ^31^, ^32^ was extended to account for the anisotropic and nonlinear biomechanical behavior of the PA vessels. The extended model was used to separate the effects of alterations in PA vessel biomechanics on pulmonary hemodynamics in a rodent model of PH. The method presented in this study provides and implements a workflow to create a fully subject‐specific in‐silico digital twin, through the integration of the vascular morphology reconstructed using x‐ray micro‐tomography^35^ of the rat lungs (Figure 1). The mechanical behavior of the arterial vessels was estimated through the ex‐vivo testing of the main PA (MPA), and the fiber architecture was compared to histological analysis. The hemodynamic data was obtained using right heart catheterization and Doppler imaging. This study is poised to assist with developing image‐based strategies to phenotype PH and to individualize and optimize patient‐specific PA remodeling‐targeted treatments aimed at normalizing MPA pressure.

## 2 METHODS

### 2.1 Experimental animals and ethics

All procedures were performed in accordance with the Guide for the Care and Use of Laboratory Animals published by the US National Institutes of Health. Approval for procedures on animal subjects was obtained from the Institutional Animal Care and Use Committee (IACUC) at the Providence VA Medical Center (IACUC 1633548).

### 2.2 Animal model of PH

A total of 22 Fischer (CDF) rats were used for this study (*n* = 11 control, *n* = 11 PH). Sixteen rats were used for ex‐vivo biomechanics (*n* = 8 control, *n* = 8 PH) with each group containing an equal number of male and female rats (*n* = 4 male, *n* = 4 female). 6 rats were used for vascular imaging (*n* = 3 control, *n* = 3 PH). All rats were male. PH was induced via an established Sugen‐hypoxia (SuHx) protocol as described by Vang et al.^36^ Briefly, PH was developed by a single subcutaneous injection of a vascular endothelial growth factor inhibitor (SU5416, 20 mg/kg body weight; APExBIO, Houston, TX) that was dissolved in a diluent (0.5% carboxymethylcellulose, 0.9% NaCl, 0.4% polysorbate 80, and 0.9% benzyl alcohol) followed by 3 weeks of normobaric hypoxia exposure (10% FIO_2_; A‐Chamber Animal Cage Enclosure with ProOx 360 High Infusion Rate O_2_ Controller, BioSpherix Ltd, Parish, NY) and subsequent housing in normoxic conditions for one additional week. The control (CTL) rats received a diluent injection and were housed in normoxic conditions until the end of the study. At the end of the study, animals were placed on a heating pad (37°C) and anesthetized with continuous isoflurane inhalation (1.5%–2%) in 100% O_2_ for the duration of the echocardiography and catheterization procedures. Transthoracic echocardiography and right heart catheterization were performed as previously described.^36^ After catheterization, the rats were euthanized under isoflurane anesthesia by exsanguination. The lungs of three rats from each group (*n* = 3 CTL, *n* = 3 PH) were isolated and used for vascular imaging, as described in the following section.

### 2.3 Vascular imaging

**FIGURE 2:**
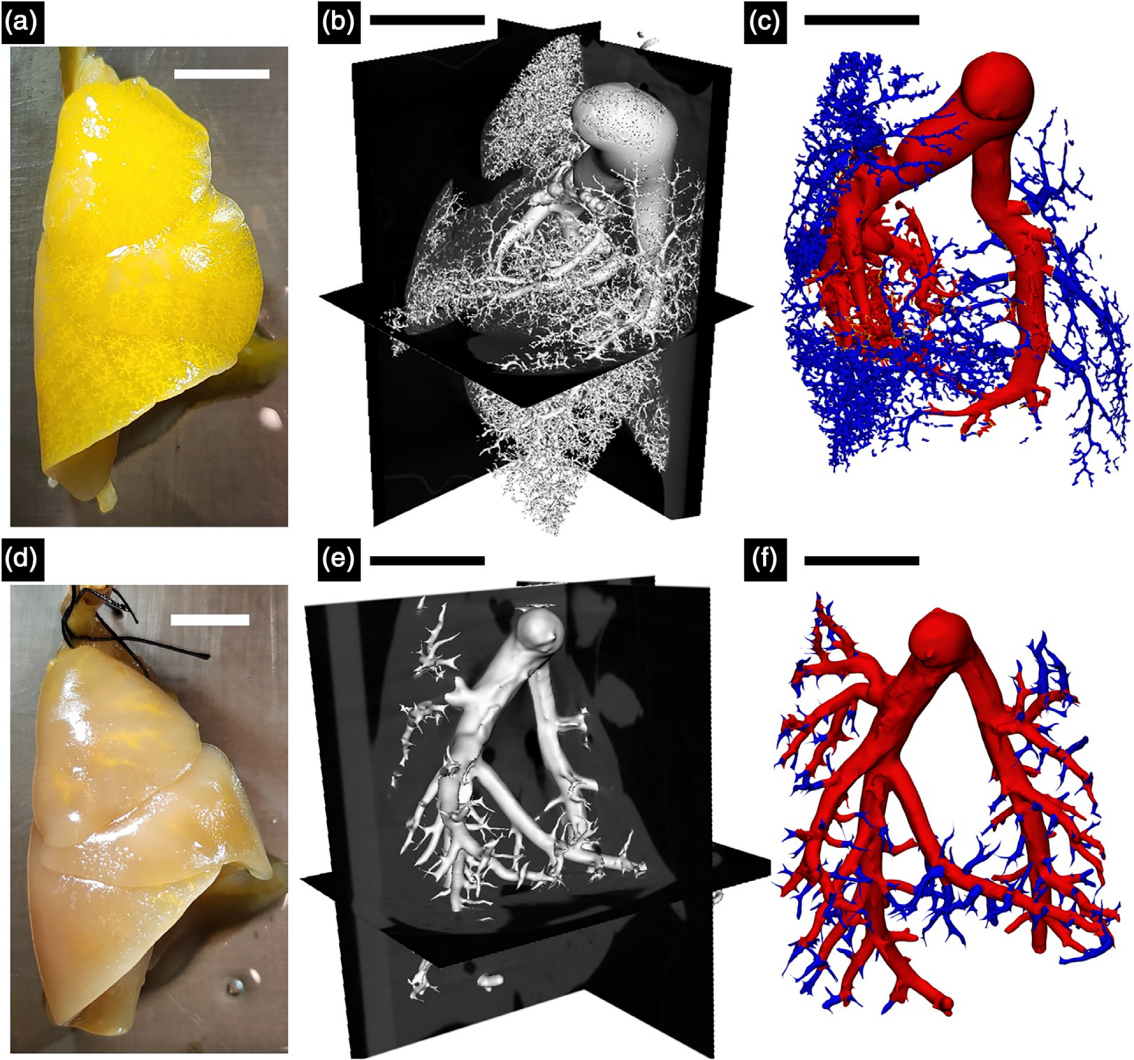
Resin‐filled lungs from (a) control (CTL) rat and (d) pulmonary hypertension (PH) rat; scale bar: 1 cm. Representative x‐ray microtomography image slices of fixed lungs, with polymer resin infused pulmonary vasculature of (b) CTL and (e) PH rat; scale bar: 5 mm. Reconstructed pulmonary vasculature of (c) CTL and (f) PH rats; scale bar: 5 mm.

The geometry of the pulmonary vasculature was obtained through the process described by Knutsen et al.^35^ Briefly, the rats were euthanized through exsanguination and placed in the supine position with the lungs and trachea exposed. The ribcage of the rat was removed to expose the heart and lungs. A 30G needle was inserted into the right ventricle through the apex, and 10^−4^ M sodium nitroprusside (SNP) in PBS was pumped in to flush the blood and dilate the pulmonary vasculature. Next, the trachea was incised, and formalin was injected using a pressure head of 20 cm H_2_O to ensure that the lungs were fixed in a pressurized and inflated state. The lungs were then excised and isolated. To cast the vasculature, a solution containing 8:1:1 of polymer: diluent: curing agent was mixed (details of the polymer compound are available in Ref. ^35^) and infused into the vasculature at a constant flow rate of 50 μL per minute through the catheter previously inserted into the apex. The compound was allowed to harden for 30–40 min at room temperature, after which the lungs were placed in 10% formalin overnight. For imaging, paraffin film was used to create a flat surface on the scanning bed, and the lungs were imaged using micro‐CT to obtain the vascular geometry of the lungs. The images were analyzed using 3D Slicer and the vascular modeling toolkit (VMTK) to obtain the length and radius of the vessels in the pulmonary vasculature (Figure 2). The VMTK toolkit was used to estimate the end points of individual vessels of the vascular tree. The endpoints of the individual vessels were used to obtain the vessel connectivity information, which, in combination with the vessel dimensions, was used to describe the 1D PA vasculature.

### 2.4 Mechanical testing and histological analysis of the PA

The MPAs of the rats, not used for vascular reconstruction (*n* = 8 CTL, *n* = 8 PH), were isolated and used to perform ex‐vivo uniaxial mechanical testing. The MPA was cut and opened along its axis to create a rectangular tissue specimen. These specimens were subjected to uniaxial mechanical testing along both the circumferential and axial (longitudinal) directions of the intact vessel using a mechanical testing machine (Cellscale Biotester; Figure 1). The specimens were stretched by 50% in both directions. The stress measured from this test was reported as the 2nd‐Piola Kirchoff (2nd‐PK) stress, and the strain was reported as Green‐Lagrange strain. Stiffness was estimated as the slope of the stress–strain curve at 60% strain (approximately equal to 50% stretch).

After biaxial testing, the PA specimens were fixed in 10% formalin and transferred to 70% ethanol after 24 h. Representative specimens that remained intact (and without large holes due to mounting) after mechanical testing were used for histological analysis (*n* = 3 CTL and *n* = 3 PH). These specimens were sectioned transmurally across the PA thickness to form 4‐μm‐thick sections. The sections were stained with Picrosirius Red (PSR) and Verhoeff–Van Gieson (VVG) in an alternating manner. The PSR and VVG stains were used to analyze the fiber architecture of collagen and elastin, respectively. The slides were imaged and used to estimate the fiber architecture through the method described in our previous studies.^11^, ^37^ Here all the positive (between 0° and 90° w.r.t. the axis of the artery) and negative (between 90° and 180° w.r.t. the axis of the artery) angles were averaged separately.

### 2.5 1D FSI model

The flow behavior in the pulmonary vasculature was simulated using a 1D FSI model adapted from Olufsen et al.^29^, ^31^, ^32^ This model describes the flow and blood vessel interaction in the pulmonary vasculature (proximal and distal). The 1D vascular tree was connected to a three‐element Windkessel model describing the flow condition in the smaller distal vessels and the capillary beds. This approach assumes that the flow is incompressible, Newtonian, laminar, and axisymmetric. Briefly, the continuity and momentum equations are integrated over the vessel's cross‐sectional area, yielding the following equations:

(1) $$\frac{\partial A}{\partial t}+\frac{\partial Q}{\partial x}=0,$$

(2) $$\frac{\partial Q}{\partial t}+\frac{\partial }{\partial x}\left(\frac{Q^{2}}{A}\right)+\frac{A}{\rho }\frac{\partial P}{\partial x}=\frac{-2\pi r}{\delta }\frac{Q}{A},$$

where *x* and *t* denote the spatial and temporal coordinates, *Q*(*x*, *t*) is the volume flow rate, *P*(*x*, *t*) is the pressure, *r*(*x, t*) is the radius of the vessel and *A*(*x*, *t*) is the cross‐sectional area. $\delta =\sqrt{\mathrm{\nu T}/2\pi }$ is the boundary layer thickness, where *T* is the time per cardiac cycle. The fluid density ρ and kinematic viscosity ν are assumed to be constant. The bifurcation of flow was based on satisfying the following conditions:

(3) $$Q_{P}=Q_{C1}+Q_{C2},$$

(4) $$P_{P}=P_{C1}=P_{C2},$$

where *Q*_*P*_ and *P*_*P*_ are the volume flow rate and pressure at the distal end of the parent vessel, *Q*_*C*1_, *P*_*C*1_, *Q*_*C*2_, *P*_*C*2_ are the volume flow rates and pressures at the proximal end of the branching child vessels. The three‐element Windkessel boundary condition to characterize the flow (*P* and *Q*) at the distal end of terminal vessels is given by an RCR circuit model:

(5) $$\frac{\mathrm{dP}\left(l,t\right)}{\mathrm{dt}}=R_{1}\frac{\mathrm{dQ}\left(l,t\right)}{\mathrm{dt}}+Q\left(l,t\right)\frac{R_{1}+R_{2}}{R_{1}R_{2}}-\frac{P\left(l,t\right)}{R_{2}C_{d}},$$

where *R*_1_ and *R*_2_ are the resistance elements, *C*_*d*_ is the compliance element, and *l* is the length of the terminal vessel where pressure is estimated. *R*_1_ and *R*_2_ were set to be equal. *R*_1_, *R*_2_, and *C*_*d*_ represent the net resistance and compliance of the smaller vessels downstream of the endpoints of the vasculature. Distal stiffness (*S*_*d*_) was defined as the inverse of distal compliance *S*_*d*_ = 1*/C*_*d*_.

The 1D model was extended to account for the PA's anisotropic and nonlinear constitutive behavior. A constitutive model of the artery wall was defined to relate the internal pressure to the lumen area. The Holzapfel–Ogden (H–O) model^38^ was used to capture both non‐linearity and anisotropy of the passive mechanical behavior of the pulmonary vasculature observed in the ex‐vivo experiments. The PA wall was assumed to be comprised of a single layer to simplify the model. The Cauchy stress state in the vessel wall is shown by **σ**. Here, $\sigma =pI+\bar{\sigma }$, where $\bar{\sigma }$ is the isochoric part of the stress tensor and *p* is the hydrostatic pressure. Equations for the isochoric stress terms in the polar coordinates are given by

(6) $${\bar{\sigma }}_{\mathrm{rr}}=c\left[\frac{2}{3}\lambda_{r}^{2}-\frac{1}{3}\left(\lambda_{\theta }^{2}+\lambda_{z}^{2}\right)\right]-\frac{4k_{1}\alpha }{3}\exp\left(k_{2}\alpha^{2}\right)\left[{\left(\lambda_{\theta }\cos\beta \right)}^{2}+{\left(\lambda_{z}\sin\beta \right)}^{2}\right],$$

(7) $${\bar{\sigma }}_{\theta \theta }=c\left[\frac{2}{3}\lambda_{\theta }^{2}-\frac{1}{3}\left(\lambda_{r}^{2}+\lambda_{z}^{2}\right)\right]+\frac{4k_{1}\alpha }{3}\exp\left(k_{2}\alpha^{2}\right)\left[{\left(\lambda_{\theta }\cos\beta \right)}^{2}-{\left(\lambda_{z}\sin\beta \right)}^{2}\right],$$

(8) $${\bar{\sigma }}_{\mathrm{zz}}=c\left[\frac{2}{3}\lambda_{z}^{2}-\frac{1}{3}\left(\lambda_{r}^{2}+\lambda_{\theta }^{2}\right)\right]+\frac{4k_{1}\alpha }{3}\exp\left(k_{2}\alpha^{2}\right)\left[{\left(\lambda_{z}\sin\beta \right)}^{2}-{\left(\lambda_{\theta }\cos\beta \right)}^{2}\right],$$

(9) $$\alpha =\lambda_{\theta }^{2}\cos^{2}\beta +\lambda_{z}^{2}\sin^{2}\beta -1,$$

where $\lambda_{r}$, $\lambda_{\theta }$, and $\lambda_{z}$ are the stretch values in the *r*, θ, and *z* directions respectively. *c*, *k*_1_, and *k*_2_ are material constants (detailed in Section S1) that were estimated using the stress‐strain data from ex‐vivo mechanical testing of the MPA. *β* is the effective fiber angle with respect to the longitudinal direction for the single‐layered tissue. Like the material parameters, *β* was estimated by fitting the stress–strain data from ex‐vivo mechanical testing of the MPA along the circumferential and longitudinal directions. In addition, *β* was compared to the histological analysis of the PA tissue specimens detailed in Section 2.3. The equilibrium equation in the radial direction was used to determine the fluid pressure:

(10) $$\frac{\partial \sigma_{\mathrm{rr}}}{\partial r}+\frac{\sigma_{\mathrm{rr}}-\sigma_{\theta \theta }}{r}=0.$$

Integrating this equation over the thickness of the vessel, assuming no pressure outside the vessel, yields the pressure in the interior of the vessel, given by

(11) $$P=\int_{r_{i}}^{r_{o}}\left(\frac{\sigma_{\theta \theta }-\sigma_{\mathrm{rr}}}{r}\right)\mathrm{dr}.$$

Here, $r_{i}$ and $r_{o}$ are the inner and outer radii of the vessel, respectively. Next, the stress terms are written in terms of the isochoric components as

(12) $$P=\int_{r_{i}}^{r_{o}}\left(\frac{{\bar{\sigma }}_{\theta \theta }-{\bar{\sigma }}_{\mathrm{rr}}}{r}\right)\mathrm{dr}.$$

Substituting Equations S.25 and S.26,

(13) $${\bar{\sigma }}_{\theta \theta }-{\bar{\sigma }}_{\mathrm{rr}}=c\left[\lambda_{\theta }^{2}-\lambda_{r}^{2}\right]+4k_{1}\alpha {\left(\lambda_{\theta }\cos\beta \right)}^{2}\exp\left[k_{2}\alpha^{2}\right].$$

The detailed derivation for Equations 6, 7, 8, and 13 can be found in Section S1 of the Supplementary Material. Non‐linear regression of the mean ex‐vivo uniaxial testing results was performed to obtain the material parameters introduced in this subsection. When the specimen was stretched along the longitudinal direction, $\sigma_{rr}={\bar{\sigma }}_{rr}+p=0$ (Equation 6) and $\sigma_{\theta \theta }={\bar{\sigma }}_{\theta \theta }+p=0$ (Equation 7) conditions were imposed to determine *p* and $\lambda_{\theta }$ in terms of the material parameters and estimate $\sigma_{\mathrm{zz}}$ (Equation 8). This process was repeated for the uniaxial test along the circumferential direction. $\sigma_{rr}={\bar{\sigma }}_{rr}+p=0$ (Equation 6) and $\sigma_{\mathrm{zz}}={\bar{\sigma }}_{zz}+p=0$ (Equation 8) conditions were imposed to determine *p* and $\lambda_{z}$ in terms of the material parameters and estimate $\sigma_{\theta \theta }$ (Equation 7). The estimated $\sigma_{\theta \theta }$ and $\sigma_{\mathrm{zz}}$ were compared against the measured values to determine the error, which was minimized using least‐squares curve fitting.

### 2.6 In‐silico experiments

#### 2.6.1 Parameterization of the model

To modulate proximal vessel stiffness, a scaling factor, termed proximal stiffness scaling (*S*_*p*_), was defined that uniformly scales material parameters *c* and *k*_1_ such that *S*_*p*_ = 1 represented mechanical behavior from control specimens (Table 1). Proximal vessel compliance is inversely proportional to *S*_p_. The three‐element Windkessel model represents the flow in the smaller distal vessels and capillary beds. In the Windkessel elements, *R*_*d*_ = *R*_1_ = *R*_2_ represented the hydraulic resistance (flow resistance), and *S*_*d*_ was taken to represent the stiffness of the elastic (distal) arterioles (Table 1). The effects of these individual remodeling mechanisms were explored, as described in the next section.

**TABLE 1:** Modeling parameters representing remodeling mechanisms.

Mechanism	Model parameter
Proximal stiffness scaling	$S_{p}$
Distal stiffness	$S_{d}$
Distal resistance	$R_{d}=R_{1}=R_{2}$

#### 2.6.2 Deconvoluting the contributions of vascular remodeling events to increased RV pulse pressure

In‐silico experiments were performed to separate the contributions of individual vascular remodeling events on the MPA pressure waveform. To begin the in‐silico simulation of the CTL rat, the PA constitutive material constants (*c*, *k*_1_, *k*_2_, *β*) were obtained by fitting the model to the ex‐vivo mechanical testing data.^39^, ^40^ In addition, *β* was also compared through the histological analysis of the PA tissue specimens detailed in Section 2.3. Since the arterial wall was assumed to be single‐layered, *β* corresponds to an effective angle used to characterize the anisotropy of the tissue. The limitations associated with this approach are detailed in Section 4.5. The values used in the in‐silico simulations were obtained for the healthy and disease specimens by fitting the model to the mean stress‐strain curves. Next, the distal stiffness (*S*_*d*_) and resistance (*R*_*d*_) were chosen such that the simulated pressure matched the measured pulse pressure at MPA (∆MPA pressure), defined as the difference between the systolic and diastolic pressures. The rate of change in RV volume was used as the flow rate at the MPA inlet, assuming no regurgitation at the tricuspid valve. The waveform was uniformly scaled such that the area under the curve (inflow volume per pulse) matched the cardiac output measured through echocardiography. This process was performed for the three PA vascular trees from the healthy CDF rats.

Next, to estimate the alteration in resistance and compliance for the in‐silico PH rat, the distal resistance (*R*_*d*_) was scaled based on the change observed in PVR from healthy to PH rats. Distal stiffness (*S*_*d*_) was scaled, and proximal stiffness scaling (*S*_*p*_) was set based on the increase in ex‐vivo mechanical stiffness. The variable ↑*S* was used to represent the combined increase of both proximal and distal stiffness. After scaling all relevant parameters, the PH model was used to estimate pressure to obtain an in‐silico prediction of ∆MPA pressure in PH using the flow waveform scaled by the average cardiac output measured in the PH animals.

#### 2.6.3 Deconvoluting the contributions of vascular remodeling events toward altered pulsatility of flow

While peak pressure is an important metric for understanding the increase in RV afterload, it is insufficient to describe the pulsatile and dynamic behavior of pressure and flow in the pulmonary vasculature and their alterations in PH. The alteration in the pulsatility of the pressure and flow waveforms is commonly analyzed using pulmonary arterial impedance (PAZ) in the frequency domain.^41^, ^42^, ^43^ The flow and pressure waveforms were converted to the frequency domain using Fourier transforms, and PAZ was calculated using the following equation:

(14) $$Z\left(\omega \right)=\frac{\left|P\left(\omega \right)\right|}{\left|Q\left(\omega \right)\right|}.$$

Two major indices of the PAZ include the impedance at 0 Hz, denoted by *Z*_0_, and the characteristic impedance, defined by *Z*_*c*_ and defined as the average impedance over a large frequency range excluding 0Hz (Figure S5 shows a representative example). Here, *Z*_*c*_ was calculated over the range of 0–250 Hz. *Z*_0_ and *Z*_*c*_ were used to understand the effect of vascular remodeling on the mean and pulsatile (oscillatory) behavior of the pressure and flow waveforms. *Z*_0_ and *Z*_*c*_ were estimated for four scenarios—control, increased vessel stiffness, increased distance resistance, and PH. Since our key objective here was to separate the effects of increased PA stiffness versus increased PA resistance on the flow pulsatility, the pulse pressure waveform was used in Equation 14 to estimate impedance. By definition, the inclusion of a diastolic pressure baseline in the pressure waveform would leave *Z*_*c*_ unaltered.

### 2.7 Statistical analysis

The experimental data was analyzed in GraphPad Prism 9. All the measured data were presented as mean ± standard error. Data from male and female rats included in the study were denoted by filled and hollow markers, respectively. Paired student *t*‐tests were performed when analyzing the statistical significance between the CTL and PH rats in Figure 4. The numerical significance values have been reported in this study.^44^

## 3 RESULTS

### 3.1 Morphological remodeling of PA vasculature

**FIGURE 3:**
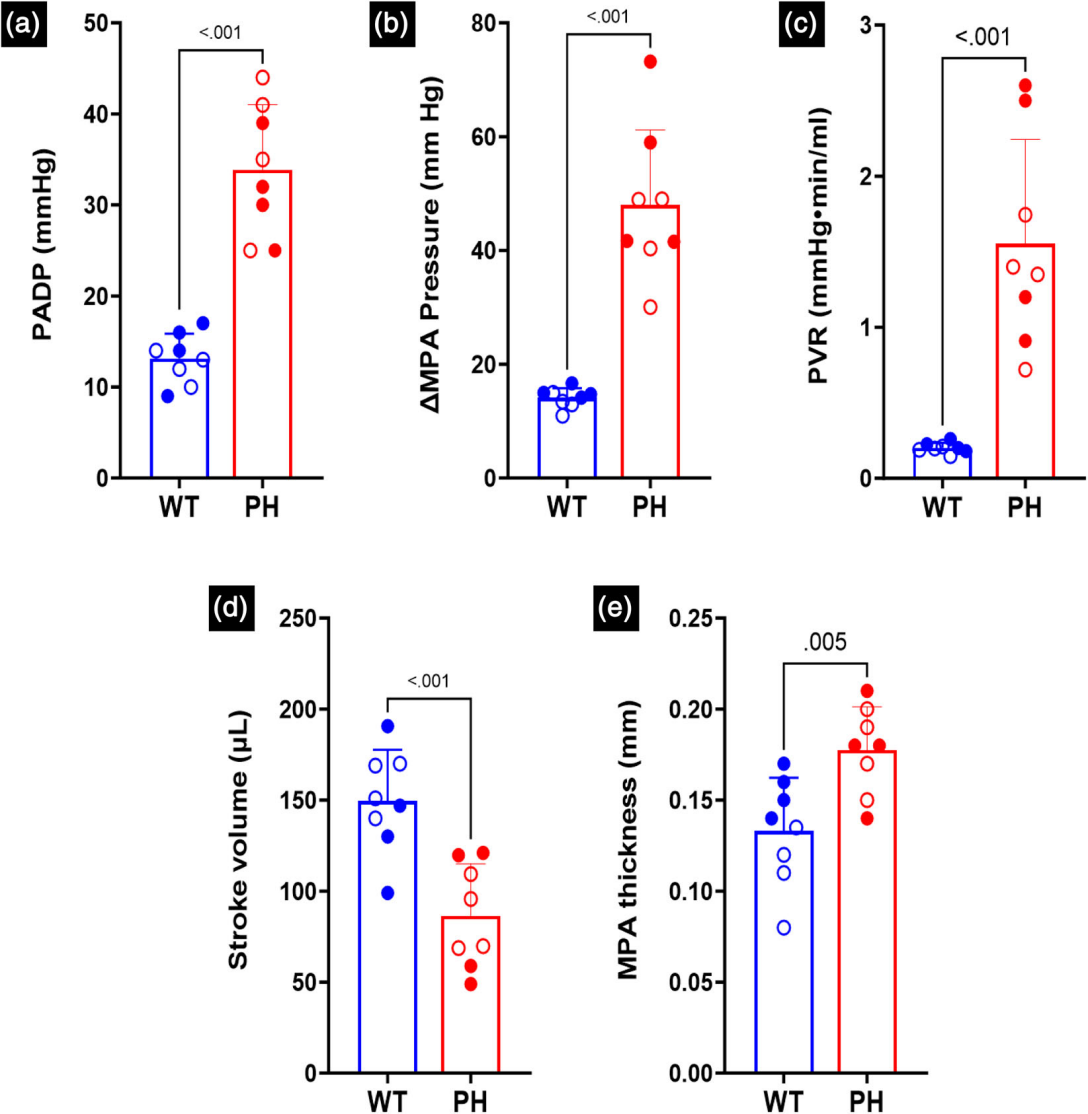
(a) Pressure in the main pulmonary artery (MPA) at diastole (PADP). (b) Difference in MPA pressure between systole and diastole (∆MPA pressure). (c) Pulmonary vascular resistance (PVR). (d) Stroke volume. (e) MPA thickness. Statistics were performed using student *t*‐tests. Male and female animals are denoted by filled and hollow markers, respectively. CTL: *n* = 8, PH: *n* = 8.

There was a decrease in the number of smaller vessels in the PH lungs after being filled with resin (Figure 2b,e). As the resin filling was performed under the same pressure and for the same amount of time, analyzing the distribution of vessel length and radius indicated an increased fraction of vessels at larger lengths and radii and a reduced fraction of vessels at smaller values of length and radius (Figure S1). This difference suggested increased resistance in the distal vessels in PH (Figure 2c,f), which was confirmed in the larger cohorts of control and PH rats with hemodynamic measurements (Figure 3). Catheterization indicated increased diastolic and systolic pressure (Figure 3a,b). The increased distal resistance observed during resin filling was reflected in the PVR measured through catheterization (Figure 3c). Stroke volume decreased (Figure 3d), which was in agreement with the literature.^10^ In addition to changes in the geometry, an increase was observed in the MPA thickness (Figure 3e).

### 3.2 Mechanical stiffening of PA tissues in PH

**FIGURE 4:**
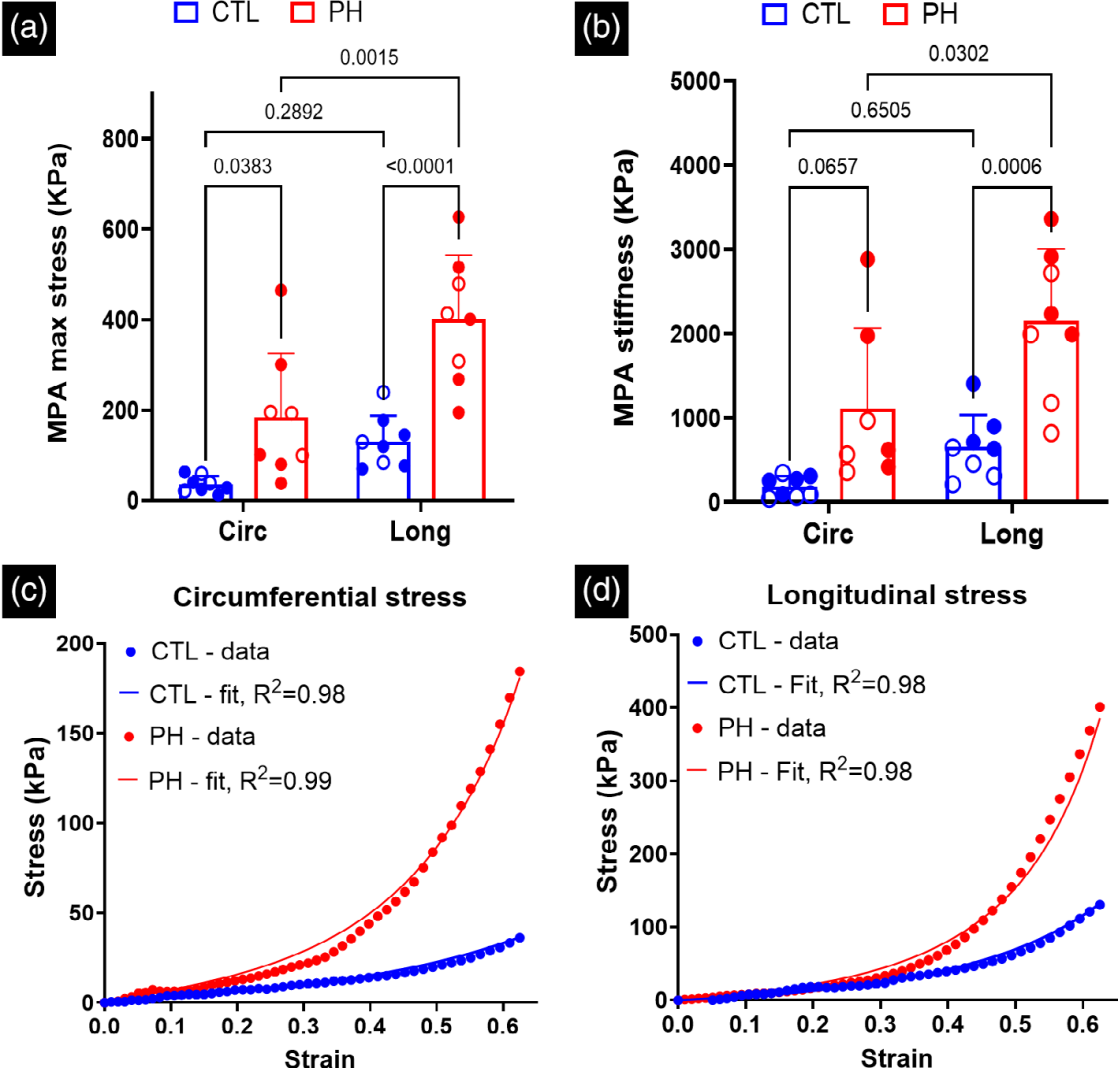
(a) Stress in the main pulmonary artery (MPA) during mechanical testing at 50% strain and (b) stiffness of the MPA at 60% strain. Fit of the non‐linear constitutive model to the PA mechanical behavior in the (c) circumferential and (d) longitudinal (axial) directions. Stress and strain denote the 2nd‐PK stress Green‐Lagrange strain, respectively. The constitutive model was fit to the mean stress–strain curve measured through uniaxial testing of PA specimens. Mean curves with error bars are presented in the Supplementary Materials. Statistics in (a), (b) were performed using two‐way ANOVA with Tukey's correction for multiple comparisons. Male and female animals are denoted by filled and hollow markers, respectively. CTL: *n* = 8, PH: *n* = 8. Circ, circumferential; Long, longitudinal.

There was a significant increase in the maximum stress (Figure 4a) and stiffness of the MPA (Figure 4b) in the longitudinal direction in the PH specimens as compared to the controls. The MPA tissue specimens of the CTL rats were biased toward the longitudinal direction (Figure S2), but the difference was not significant. The MPA specimens of the PH rats were further biased toward the longitudinal direction (Figure S2), with this change being significant when comparing the maximum stress (Figure 4a) and near‐significant when comparing the stiffness (Figure 4b). The non‐linear constitutive model proposed in this study was able to closely capture the behavior of the PA specimens observed during ex‐vivo uniaxial testing (Figure 4c,d and Table 2).

**TABLE 2:** Parameters of the constitutive model obtained by fitting to the mean stress–strain curve of each group.

Material Parameter	WT	PH
*c* (kPa)	10	20
*k*_1_ (kPa)	100.2	310
*k*_2_	5.3	15.2
*β* (°)	48.2	46.2

*Note*: Variation of material parameters obtained by fitting to individual curves has been presented in Table S1.

### 3.3 Histological analysis

Histological analysis was performed to compare the angle *β*, estimated through fitting the constitutive model to ex‐vivo data, with fiber angle data acquired from histology. The PSR and VVG stains were used to extract the orientation of the collagen and elastin fibers, respectively (representative images presented in Figure S3). The analysis indicated that *β* captured an average of the fiber angle, including both collagen and elastin (Table 3). The values of *β* estimated from fitting the model to the ex‐vivo mechanical testing data were used for the 1D FSI simulations.

**TABLE 3:** Collagen and elastin fiber angle in the PA tissue specimens.

Group	+ve angle collagen (°)	−ve angle collagen (°)	+ve angle elastin (°)	−ve angle elastin (°)
Control	51.45 ± 24.97	−56.55 ± 25.32	53.73 ± 21.83	−46.36 ± 23.78
PH	48.24 ± 16.79	−49.82 ± 19.25	47.76 ± 26.84	−44.59 ± 23.58

*Note*: Angle was estimated with respect to the axis of the intact vessel. *n* = 3. +ve, positive; −ve, negative.

### 3.4 Relationship between vascular remodeling and pulmonary hemodynamics

**FIGURE 5:**
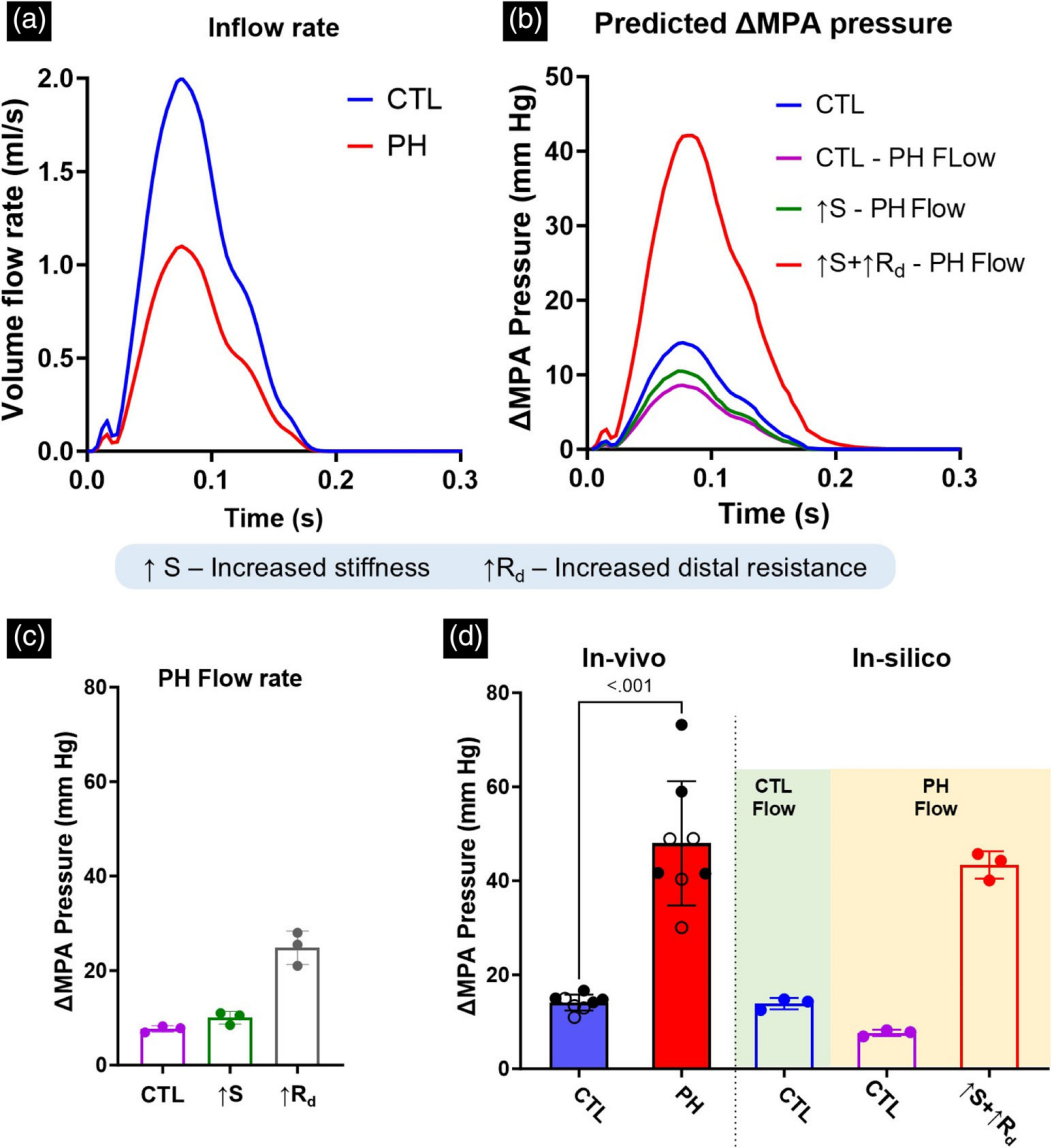
(a) Measured main pulmonary artery (MPA) flow profile in the control (CTL) and pulmonary hypertension (PH) rats used in the in‐silico model. (b) The simulated pressure waveform obtained from the in‐silico model for the MPA after altering flow and remodeling parameters. (c) Simulated changes in ∆MPA pressure as a result of individual remodeling parameters. (d) The ∆MPA pressure obtained through in‐vivo measurements and in‐silico simulations. Male and female animals are denoted by filled and hollow markers, respectively. For in‐vivo measurements: CTL, *n* = 8; PH, *n* = 8. For in‐silico simulations, *n* = 3 control vascular geometries were used. ∆MPA Pressure is the difference in the MPA pressure between systole and diastole.

The measured volume flow rates (Figure 5a) were used as inputs to investigate the cumulative effects of the PA remodeling events on ∆MPA pressure. There were alterations observed in both the maximum value and relaxation behavior of the pressure waveform (Figure 5b). When the model resistance and stiffness were individually modified to match the values observed in the PH rats, the increase in distal resistance had the largest effect toward increased ∆MPA pressure (Figure 5c). The observed vessel thickening (Figure 3e) was incorporated into the increased PA stiffness. Increased vessel stiffness led to smaller and relatively equal increases in ∆MPA pressure (Figure 5c). In addition, increased stiffness did not cause significant changes to the qualitative behavior of the pressure waveform (Figure S4). However, increased resistance caused a delay between the peak of the volume flow rate and the pressure waveform and resulted in a significantly slower drop in pressure from the peak (Figure S4). Next, the cumulative effects of the remodeling events were calculated (Figure 5d). Expectedly, incorporating decreased flow rate (CTL ‐ PH Flow; Figure 5d) led to a decrease in ∆MPA pressure. Increased stiffness (↑S; Figure 5d) led to small increases in ∆MPA pressure, while adding the effect of increased resistance caused a significant increase in ∆MPA pressure (↑S+↑R_d_; Figure 5d). However, comparing the individual and cumulative effects of the PA remodeling events indicated that a notable portion of the increase in ∆MPA pressure originated from the interactions between these two events (Figure S4). When the resistance, stiffness, and volume flow rate all matched the corresponding values in PH rats, the ∆MPA pressure value became comparable to the experimentally observed ∆MPA pressure values in the PH rats (↑S+↑R_d_ PH Flow; Figure 5d).

**FIGURE 6:**
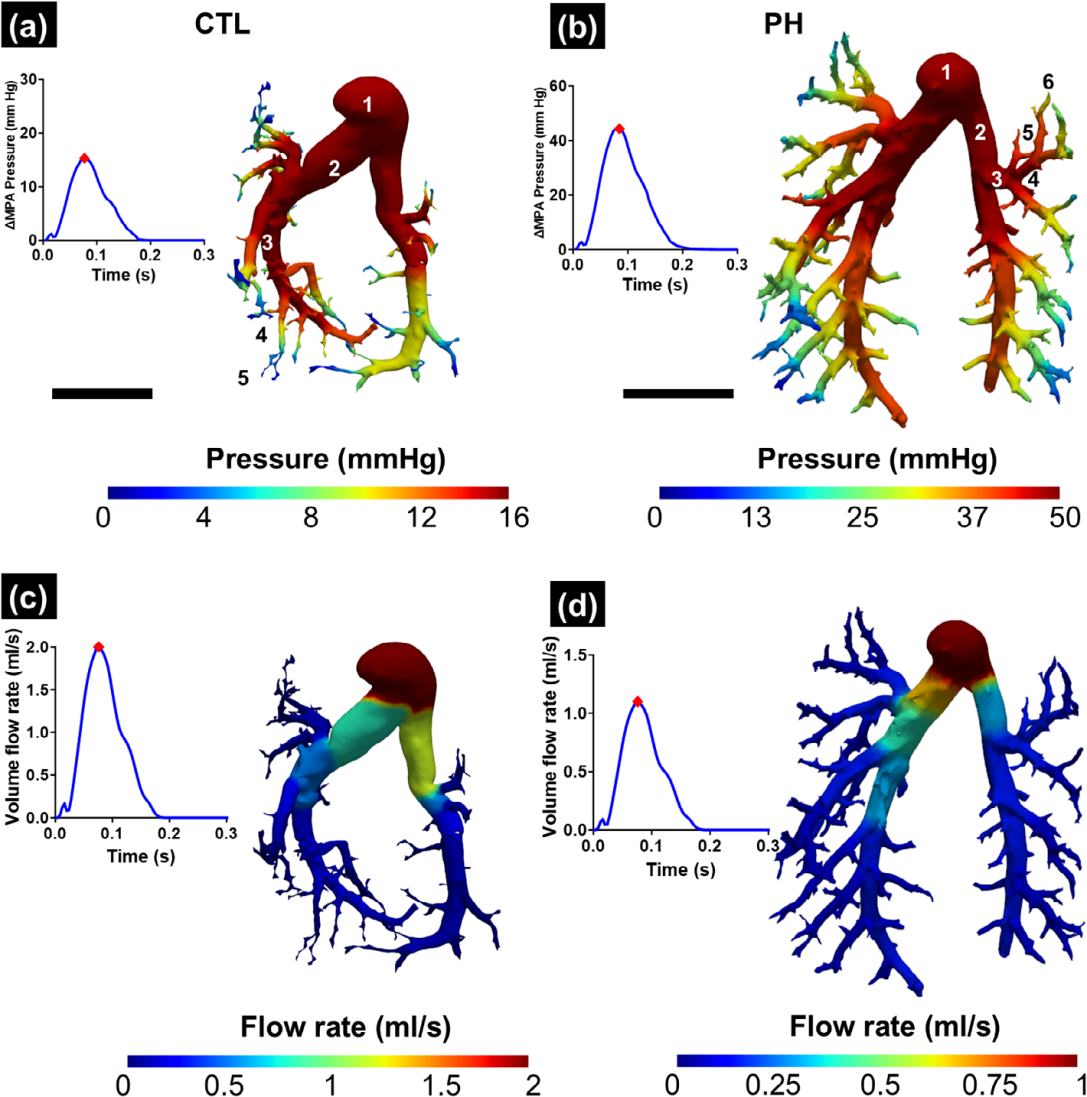
Variation of pressure in the pulmonary vasculature in (a) control (CTL) and (b) pulmonary hypertension (PH) rats with the corresponding time point marked on the pressure–time plot. Variation of flow rate in the pulmonary vasculature in (c) CTL and (d) PH rats. Results from the 1D FSI simulation were mapped onto the 3D geometry obtained from segmentation with the corresponding time point marked on the flow–time plot. Representative branching generations of the vessel have been labeled in (a) and (b). ∆MPA Pressure: difference in the MPA pressure between systole and diastole. Both scale bars represent 5 mm.

When investigating the downstream pressure drop in the complete vasculature, a sharp decrease was observed in the pressure after 3–4 generations in both the healthy and PH geometries (Figure 6a,b). The flow rate in the vasculature split at the branching points in the same ratio as the lumen area of the daughter vessels (Figure 6c,d). This led to the two primary branches carrying the majority of the flow and a significant decrease when the flow reached the smaller vessels.

### 3.5 Effect of PA remodeling on PA impedance

**FIGURE 7:**
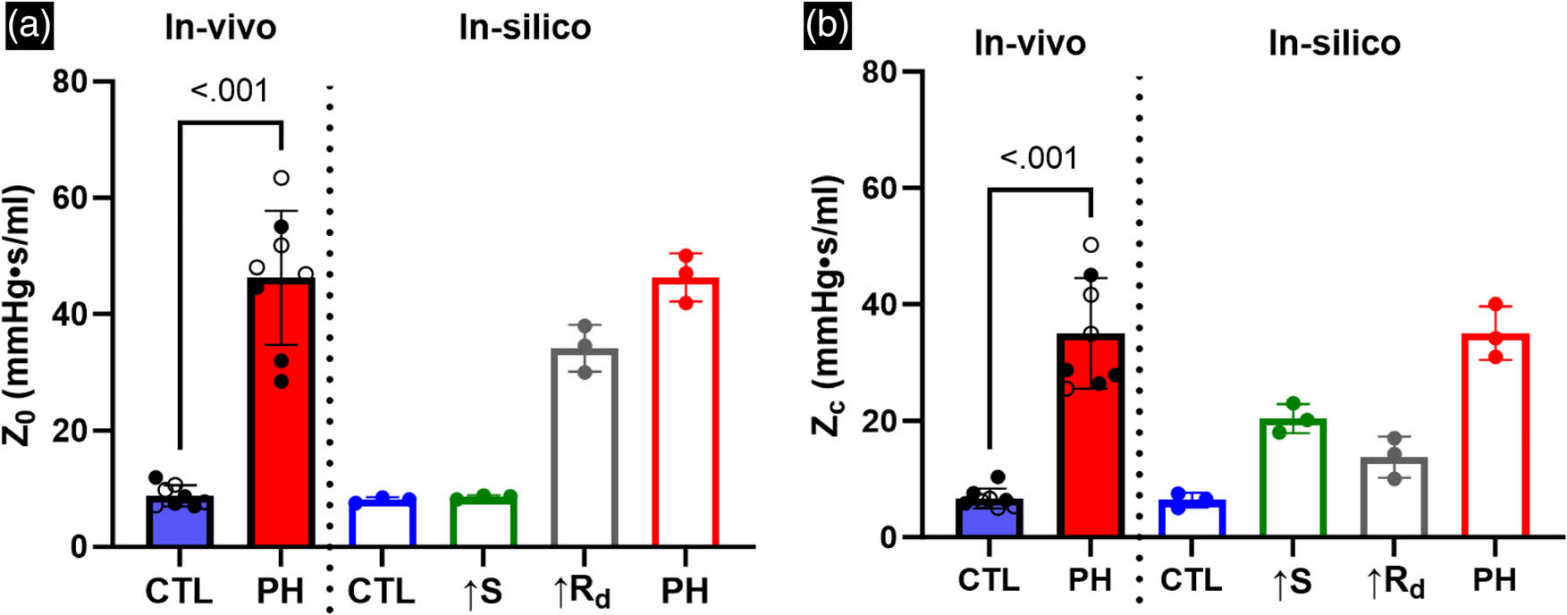
(a) Variation of 0 Hz PA impedance (*Z*_0_) due to PA remodeling events. (b)Variation of characteristic PA impedance (*Z*_c_) due to PA remodeling events. Male and female animals are denoted by filled and hollow markers, respectively. CTL, *n* = 8; PH, *n* = 8. For in‐silico simulations, *n* = 3 control vascular geometries were used. Statistical significance was estimated through Student's *t*‐test to compare the in‐vivo impedance values.

*Z*_0_ and *Z*_*c*_ were estimated from the variation of PAZ with frequency (Figure S5). Frequency domain analysis indicated that increased resistance was the dominant contributor toward *Z*_0_ (Figure 7a), with *Z*_0_ due to increased resistance being 82.7% of the *Z*_0_ in PH. In contrast, increased stiffness had a larger contribution toward *Z*_*c*_ compared to increased resistance (Figure 7b), with *Z*_*c*_ due to increased resistance being 41% of the *Z*_*c*_ in PH. The impedance frequency analysis also indicated an interaction between increased stiffness and resistance, with the *Z*_0_ and *Z*_*c*_ values estimated from the in‐silico PH simulation not being equal to the arithmetic sum of the impedance values estimated in the cases of the individual remodeling events. In addition, *Z*_0_ and *Z*_*c*_ estimated through in‐silico modeling closely matched the mean *Z*_0_ and *Z*_*c*_ values estimated through catheterization (Figure 7a,b).

### 3.6 Verification of 1D FSI model and the effect of non‐linear vessel behavior

The model was verified by simulating a constant volume flow rate through a near‐rigid tube (detailed in Section S7 in the supplementary material). The Windkessel resistance values were set to 0 (i.e., zero outflow pressure). We observed that the pressure contour estimated by the 1D FSI model matched the result provided by a commercial computational fluid dynamics software (3D FSI using ANSYS Fluent and Structural; Figure S6A,B). We also compared the effect of linear and non‐linear vessel behavior on the expansion of the lumen area. We observe that the behavior is similar under small values of area deformation, but the non‐linear vessel behavior presented in Section 2.4 leads to significantly larger pressure values at large values of lumen area deformation (Figure S7). This indicates a need for a significant increase in RV afterload to maintain similar flow behavior in the pulmonary vasculature.

## 4 DISCUSSION

### 4.1 Deconvoluting the effects of vascular remodeling events on MPA pressure

We have presented a method to investigate the subject‐specific contribution of increased resistance and vessel stiffness to the increased MPA pressure in a PH model of rats. The results of this study indicate that the remodeling mechanisms have a complex interplay and that summing the individual effects of each mechanism fails to fully capture the final PH state. This highlights the utility of the model presented in this study in investigating and estimating the effect of each mechanism, as well as their interactions with each other. Under a fixed input flow rate at the MPA, each individual vascular remodeling event resulted in an increase in ∆MPA pressure. This indicates that increased distal resistance and increased stiffness in both the proximal and distal vessels each contribute to increased ∆MPA pressure. Increased resistance had the greatest effect on increasing the ∆MPA pressure. This is consistent with the use of PVR as a key longitudinal metric of PH. Increased proximal and distal stiffness affected pressure similarly; their effect on pressure was smaller than that of resistance.

As previously stated, the final PH ∆MPA pressure was not the result of the sum of the changes from each individual mechanism, suggesting a significant interaction taking place between mechanisms. Interactions between remodeling events were most evident in the relationships between proximal stiffness and other mechanisms. Increased proximal stiffness had greater effects on peak ∆MPA pressure when present in conjunction with increased resistance. Indeed, these results highlight the complex nature of post‐PH remodeling and point toward a need for the continued exploration of patient‐specific modeling approaches to identify the most effective therapeutic plan in each case.

### 4.2 Effect of vascular remodeling on pulsatility of flow

Notable changes were observed in the pressure waveform due to the remodeling events, given a fixed volume flow rate waveform. These variations in the pressure waveform were reflected in the 0Hz (*Z*_0_) and characteristic (*Z*_*c*_) PA impedance values estimated through Fourier analysis. Studies have indicated that PA impedance is a more comprehensive biomarker than PVR due to its ability to capture both static and pulsatile components of the pressure and flow waveforms.^43^, ^45^ The study by Hunter et al.^45^ indicated that *Z*_0_ demonstrated a significant correlation with PVR, while *Z*_*c*_ correlated with decreased vessel compliance.

Our results are in agreement with these studies, with increased resistance being the dominant contributor toward increased *Z*_0_ and increased stiffness being the major contributor toward increased *Z*_*c*_. Also, studies have reported that PA impedance measurements correlate better with patient outcomes in PH,^43^, ^45^, ^46^, ^47^ corroborating that peak pressure is insufficient to understand the degree of remodeling and its effect on the pulmonary circulation.

In addition, the pressure waveform had several qualitative variations due to the vascular remodeling events. In the case of increased flow resistance, there was a delay between the peaks in volume flow rate and pressure. In addition, increased resistance also resulted in a significantly slower pressure drop, with pressure requiring the duration of one volume flow pulse to drop to EDP. These variations are hypothesized to be a result of a combination of pruning (reduction in the number of arterioles and alveolar capillaries)^1^, ^2^ and increased resistance in the distal vessels impeding fluid flow, leading to the accumulation of fluid. This accumulated fluid slowly exits the vessel, thus leading to an “uncoupled" behavior with the volume flow rate waveform. However, incorporating increased vessel stiffness and resistance reduced the delay between pressure and volume flow rate waveforms and speed up the rate of pressure drop. This was expected as the increased vessel stiffness prevented excessive vessel dilation, effectively forcing the fluid downstream.

Finally, we note that, since determining the contribution of PA stiffening to the characteristic impedance (*Z*_*c*_) was the focus of the impedance analysis, pulse pressure was used to calculate the impedance. Adding a constant value (PA diastolic pressure) to the pulse pressure will only affect *Z*_0_ and keep *Z*_*c*_ unchanged, and, therefore, it will not change our findings regarding the role of resistance vs. stiffening in impedance. However, using full pressure instead of the pulse pressure will only increase *Z*_0_ values and thus lead to a larger contrast between *Z*_0_ and *Z*_*c*_. Such larger contrasts will be in agreement with existing studies indicating that *Z*_0_ values tend to be larger than *Z*_*c*_ values, potentially by one order of magnitude.^43^, ^48^

### 4.3 Capturing PA remodeling using a non‐linear and anisotropic material model

In this work, the H‐O (non‐linear anisotropic) constitutive model was used to describe the mechanical behavior of the arterial vessels. To our knowledge, this is the first example of the incorporation of a non‐linear anisotropic material model that is expressly designed to model arterial behavior into a 1D FSI modeling approach. While vessel behavior has been shown to be non‐linear, linear constitutive models are commonly used for their simplicity. The primary difference between linear and non‐linear vessel behavior is the capability of non‐linear behavior to capture the significantly increased pressure at large values of lumen area expansion. Vascular remodeling events, such as narrowing of the distal vasculature, arterial hypertrophy, and pruning, lead to a reduction in the lumen cross‐sectional area, leading to a larger area expansion under similar vascular blood flow. The predicted pressure as a result of larger area deformation of the vessels is not capable of being accurately captured by a linear behavior regime and is more accurately represented by a non‐linear vessel model. In addition, the increased arterial wall stiffness leads to increased stiffness of the arteries and arterioles. This increased stiffness, combined with the decreased lumen area, will lead to an increased flow rate through the arterioles and capillaries, reducing oxygen diffusion into and carbon dioxide out of the bloodstream. This effect manifests as shortness of breath, which has been reported to be a symptom of PH by several studies.^17^, ^49^, ^50^

In addition to non‐linear mechanical behavior, the incorporation of material anisotropy is also essential when investigating the vascular remodeling observed in PH. Our results indicated that the stiffening of the vessel walls is not uniform in the circumferential and longitudinal directions. The MPA stiffness from healthy rats was mildly biased toward the circumferential direction, and this bias shifted towards the longitudinal (axial) direction in PH rats. These results indicate fiber re‐orientation in the vessels due to PH and emphasize the need for an anisotropic material model. The alteration in vessel wall anisotropy also highlights a potential opportunity to introduce longitudinal dependence on pressure in the solid domain of the 1D FSI model.

### 4.4 Implications for potential clinical application

A combination of in‐vivo and ex‐vivo measurements that characterize the increase in MPA pressure and vascular impedance due to PH was incorporated into an in‐silico model. Then, *hypothetical states* were generated in silico to analyze the isolated effect of individual remodeling mechanisms on ∆MPA pressure and the characteristic impedance. This modeling approach can enable an improved subject‐specific understanding of the interaction between vascular remodeling events and between individual events on the RV and PA sides.^51^, ^52^ The approach presented in this study could be translated to the clinical setting to enable patient‐specific simulation of the full pulmonary vasculature, representing a critical step toward optimizing clinical intervention strategies. However, the data acquisition methods used to obtain experimental measurements would require modifications for application in the clinic. To apply this method to human patients in clinical settings, data would need to be acquired via non‐invasive methods, such as medical imaging, or minimally invasive methods, such as catheterization. The primary parameters required for a patient‐specific simulation are the morphology of the pulmonary vasculature, pulmonary vascular compliance and resistance, and the pulmonary flow rate and pressure. The structure of the pulmonary vasculature can be reconstructed through pulmonary angiography. The vessel compliance, vascular resistance, pulmonary blood flow rate, and pressure can be estimated through the measurement of PAC and PVR using echocardiography and catheterization, respectively.^53^, ^54^ This data, combined with the approach presented in this study, could potentially be used to dissect the contribution of the different remodeling events on a patient‐specific basis, facilitating individualized risk stratification and leading to improved clinical therapeutic strategies.

### 4.5 Limitations

In this study, the PA radius was measured from *μ*CT imaging. This radius may not exactly reflect the radius of the vessels in vivo during diastole due to two factors. Firstly, the fixation was performed at elevated pressure (15 mmHg)^35^ to allow for the expansion and imaging of smaller vessels. This can lead to the over‐expansion of vessels. However, this pressure was similar to the mean pulmonary arterial diastolic pressure (PADP) for the control specimens (13.1 mmHg). The comparable values between PADP and the fixation pressure suggest that the major arteries will not be significantly expanded beyond their diastolic radii when perfused at a pressure head of 15 mmHg. In addition, when estimating the radius change due to a lumen pressure of 15 mmHg, the increase in radius was 5.25% and 2.5% for the control and PH specimens, respectively (Figure S7 in the supplement). Thus, the error of deviation from diastolic radii can range from negligible (given that the diastolic pressure is comparable to 15 mmHg) to at most 5.25%. Hence, we are confident that the measured radius is not significantly altered due to the filling process. Secondly, the measured radius from *μ*CT may be susceptible to errors from the resin filling, imaging, and post‐processing. This factor will primarily affect smaller vessels, which may not be fully infused with resin, and the radius may be incorrect due to imaging resolution and processing artifacts. Additionally, one of the limitations associated with the choice to use 1D FSI modeling for this study is the inability of such models to investigate complex fluid dynamics, such as vorticity at branch points and wall shear stress behavior. Vorticity and wall shear stress can lead to the proliferation of endothelial cells in the inner wall of the vessels, resulting in maladaptation. However, a 1D FSI model was chosen since the primary objective of this study was to investigate the alterations of pulmonary arterial pressure and pulsatility of blood flow. The use of a 1D FSI model was sufficient for the accurate estimation of these parameters.

Estimation of the material parameters for the pulmonary tissue from ex‐vivo mechanical testing could potentially introduce uncertainties in parameter values through factors such as tissue handling, inherent variability, and noise introduced during the measurement of force in the tensile tests. We adhered to strict measurement protocols to minimize the errors in these processes. In addition, we used average values utilizing eight specimens per group (with equal numbers of male and female rats) to reflect population‐average behaviors in our parameter estimation. In future investigations, we will expand this study using a larger cohort of rats. In this study, the effective fiber angle estimated by fitting the constitutive model to the ex‐vivo uniaxial testing data was compared against histological analysis of the PA tissue. One factor limiting the accuracy of the fiber angles estimated from the histological analysis was the presence of larger holes in some thinner specimens after mechanical testing. Future studies will use a larger cohort to ensure a more accurate estimation of collagen and elastin fiber angles from tissue specimens.

## 5 CONCLUSIONS

In this study, we presented a computational approach to separate the effects of multiple vascular remodeling events in the pulmonary arteries toward the altered MPA pressure amplitude and pulsatility associated with PH. We combined in‐silico modeling with in‐vivo and ex‐vivo measurements from healthy and diseased rats to generate in‐silico hypothetical states of the increase in MPA pressure caused by an isolated remodeling event. The results of our study indicated that increased vascular resistance is the dominant contributor toward increased pressure amplitude, while increased vessel stiffness is the main contributor toward altered pulsatility. The method presented in this study offers a rigorous tool to perform systematic computational phenotyping of PH, thus improving individualized treatment based on quantifying the contribution of vascular remodeling events to pulmonary hemodynamic dysfunction.

## CONFLICT OF INTEREST STATEMENT

The authors declare no conflict of interest.

## Supporting information


**Data S1:** Supporting Information.

## Data Availability

The data that support the findings of this study are available from the corresponding author upon reasonable request.

## References

[1] Hoeper MM , Ghofrani HA , Grünig E , Klose H , Olschewski H , Rosenkranz S . Pulmonary hypertension. Deutsches Arzteblatt Int. 2017;114:73‐84. 10.3238/arztebl.2017.0073 PMC5331483 28241922

[2] Vanderpool RR , Kim AR , Molthen R , Chesler NC . Effects of acute rho kinase inhibition on chronic hypoxia‐induced changes in proximal and distal pulmonary arterial structure and function. J Appl Physiol. 2011;110:188‐198. 21088209 10.1152/japplphysiol.00533.2010 PMC3253002

[3] Hassoun PM , Taichman DB . Pulmonary arterial hypertension. N Engl J Med. 2021;385:2361‐2376. 34910865 10.1056/NEJMra2000348

[4] Ruopp NF , Cockrill BA . Diagnosis and treatment of pulmonary arterial hypertension. JAMA. 2022;327:1379. 35412560 10.1001/jama.2022.4402

[5] Jang S , Vanderpool RR , Avazmohammadi R , et al. Biomechanical and hemodynamic measures of right ventricular diastolic function: translating tissue biomechanics to clinical relevance. J Am Heart Assoc. 2017;6:e006084. 28899895 10.1161/JAHA.117.006084 PMC5634275

[6] Avazmohammadi R , Mendiola EA , Soares JS , et al. A computational cardiac model for the adaptation to pulmonary arterial hypertension in the rat. Ann Biomed Eng. 2019;47:138‐153. 30264263 10.1007/s10439-018-02130-y PMC6318016

[7] Avazmohammadi R , Hill M , Simon M , Sacks M . Transmural remodeling of right ventricular myocardium in response to pulmonary arterial hypertension. APL Bioeng. 2017;1:016105. 30417163 10.1063/1.5011639 PMC6224170

[8] Kwan ED , Vélez‐Rendón D , Zhang X , et al. Distinct time courses and mechanics of right ventricular hypertrophy and diastolic stiffening in a male rat model of pulmonary arterial hypertension. Am J Phys Heart Circ Phys. 2021;321:H702‐H715. 10.1152/ajpheart.00046.2021 PMC8794227 34448637

[9] Sharifi Kia D , Kim K , Simon MA . Current understanding of the right ventricle structure and function in pulmonary arterial hypertension. Front Physiol. 2021;12:641310. 34122125 10.3389/fphys.2021.641310 PMC8194310

[10] Mendiola EA , da Silva Gonçalves Bos D , Leichter DM , et al. Right ventricular architectural remodeling and functional adaptation in pulmonary hypertension. Circ Heart Fail. 2023;16:e009768. 36748476 10.1161/CIRCHEARTFAILURE.122.009768 PMC9974595

[11] Neelakantan S , Vang A , Mehdi RR , et al. Right ventricular stiffening and anisotropy alterations in pulmonary hypertension: mechanisms and relations to right heart failure. J Am Heart Assoc. 2025;14:e037126. 40008537 10.1161/JAHA.124.037126 PMC12132615

[12] Galiè N , Humbert M , Vachiery JL , et al. 2015 ESC/ERS guidelines for the diagnosis and treatment of pulmonary hypertension: the joint task force for the diagnosis and treatment of pulmonary hypertension of the European Society of Cardiology (ESC) and the European Respiratory Society (ERS): endorsed by: Association for European Paediatric and Congenital Cardiology (AEPC), International Society for Heart and Lung Transplantation (ISHLT). Eur Heart J. 2015;37:67‐119. 26320113 10.1093/eurheartj/ehv317

[13] D'Alonzo GE , Barst RJ , Ayres SM , et al. Survival in patients with primary pulmonary hypertension. Ann Intern Med. 1991;115:343‐349. 1863023 10.7326/0003-4819-115-5-343

[14] Benza RL , Miller DP , Barst RJ , Badesch DB , Frost AE , McGoon MD . An evaluation of long‐term survival from time of diagnosis in pulmonary arterial hypertension from the reveal registry. Chest. 2012;142:448‐456. 22281797 10.1378/chest.11-1460

[15] McGoon MD , Benza RL , Escribano‐Subias P , et al. Pulmonary arterial hypertension: epidemiology and registries. J Am Coll Cardiol. 2013;62:D51‐D59. 24355642 10.1016/j.jacc.2013.10.023

[16] Sitbon O , Brenot F , Denjean A , et al. Inhaled nitric oxide as a screening vasodilator agent in primary pulmonary hypertension. A dose–response study and comparison with prostacyclin. Am J Respir Crit Care Med. 1995;151:384‐389. 7842196 10.1164/ajrccm.151.2.7842196

[17] Galie N , Hoeper MM , Humbert M , et al. Guidelines for the diagnosis and treatment of pulmonary hypertension: the task force for the diagnosis and treatment of pulmonary hypertension of the european society of cardiology (esc) and the european respiratory society (ers), endorsed by the international society of heart and lung transplantation (ishlt). Eur Heart J. 2009;30:2493‐2537. 19713419 10.1093/eurheartj/ehp297

[18] Avazmohammadi R , Mendiola EA , Li DS , Vanderslice P , Dixon RAF , Sacks MS . Interactions between structural remodeling and hypertrophy in the right ventricle in response to pulmonary arterial hypertension. J Biomech Eng. 2019;141:910161‐9101613. 10.1115/1.4044174 PMC6807999 31260516

[19] Odeigah OO , Valdez‐Jasso D , Wall ST , Sundnes J . Computational models of ventricular mechanics and adaptation in response to right‐ventricular pressure overload. Front Physiol. 2022;13:948936. 36091369 10.3389/fphys.2022.948936 PMC9449365

[20] Li DS , Mendiola EA , Avazmohammadi R , Sachse FB , Sacks MS . A multi‐scale computational model for the passive mechanical behavior of right ventricular myocardium. J Mech Behav Biomed Mater. 2023;142:105‐788. 10.1016/j.jmbbm.2023.105788 PMC10357348 37060716

[21] Kheyfets VO , O'Dell W , Smith T , Reilly JJ , Finol EA . Considerations for numerical modeling of the pulmonary circulation—a review with a focus on pulmonary hypertension. J Biomech Eng. 2013;135:61011‐61015. 23699723 10.1115/1.4024141 PMC3705788

[22] Kheyfets VO , Rios L , Smith T , et al. Patient‐specific computational modeling of blood flow in the pulmonary arterial circulation. Comput Methods Prog Biomed. 2015;120:88‐101. 10.1016/j.cmpb.2015.04.005 PMC4441565 25975872

[23] Kong F , Kheyfets V , Finol E , Cai X . An efficient parallel simulation of unsteady blood flows in patient‐specific pulmonary artery. Int J Numer Methods Biomed Eng. 2018;34:e2952. 10.1002/cnm.2952 29245182

[24] Zambrano BA , McLean NA , Zhao X , et al. Image‐based computational assessment of vascular wall mechanics and hemodynamics in pulmonary arterial hypertension patients. J Biomech. 2018;68:84‐92. 29310945 10.1016/j.jbiomech.2017.12.022 PMC5783768

[25] Kong F , Kheyfets V , Finol E , Cai X . Simulation of unsteady blood flows in a patient‐specific compliant pulmonary artery with a highly parallel monolithically coupled fluid–structure interaction algorithm. Int J Numer Methods Biomed Eng. 2019;35:e3208. 10.1002/cnm.3208 30989794

[26] Shavik SM , Tossas‐Betancourt C , Figueroa CA , Baek S , Lee LC . Multiscale modeling framework of ventricular‐arterial bi‐directional interactions in the cardiopulmonary circulation. Front Physiol. 2020;11:2. 32116737 10.3389/fphys.2020.00002 PMC7025512

[27] Cuomo F et al. Sex‐dependent differences in central artery haemodynamics in normal and fibulin‐5 deficient mice: implications for ageing. Proc R Soc Lond A Math Phys Eng Sci. 2019;475:20180076. 10.1098/rspa.2018.0076 PMC6364598 30760948

[28] Hopper SE , Weiss D , Mikush N , et al. Central artery hemodynamics in angiotensin ii‐induced hypertension and effects of anesthesia. Ann Biomed Eng. 2024;52:1051‐1066. 38383871 10.1007/s10439-024-03440-0 PMC11418744

[29] Olufsen MS , Peskin CS , Kim WY , Pedersen EM , Nadim A , Larsen J . Numerical simulation and experimental validation of blood flow in arteries with structured‐tree outflow conditions. Ann Biomed Eng. 2000;28:1281‐1299. 11212947 10.1114/1.1326031

[30] Qureshi MU , Colebank MJ , Paun LM , et al. Hemodynamic assessment of pulmonary hypertension in mice: a model‐based analysis of the disease mechanism. Biomech Model Mechanobiol. 2018;18:219‐243. 30284059 10.1007/s10237-018-1078-8

[31] Colebank MJ , Paun LM , Qureshi MU , et al. Influence of image segmentation on one‐dimensional fluid dynamics predictions in the mouse pulmonary arteries. J R Soc Interface. 2019;16:20190284. 10.1098/rsif.2019.0284 PMC6833336 31575347

[32] Colebank MJ , Qureshi MU , Rajagopal S , Krasuski RA , Olufsen MS . A multiscale model of vascular function in chronic thromboembolic pulmonary hypertension. Am J Phys Heart Circ Phys. 2021;321:H318‐H338. 10.1152/ajpheart.00086.2021 PMC8410122 34142886

[33] Pfaller MR , Pham J , Verma A , et al. Automated generation of 0d and 1d reduced‐order models of patient‐specific blood flow. Int J Numer Methods Biomed Eng. 2022;38:e3639. 10.1002/cnm.3639 PMC9561079 35875875

[34] Gharahi H , Filonova V , Mullagura HN , Nama N , Baek S , Figueroa CA . A multiscale framework for defining homeostasis in distal vascular trees: applications to the pulmonary circulation. Biomech Model Mechanobiol. 2023;22:971‐986. 36917305 10.1007/s10237-023-01693-7

[35] Knutsen RH , Gober LM , Sukinik JR , et al. Vascular casting of adult and early postnatal mouse lungs for micro‐CT imaging. JoVE. 2020;e61242. 10.3791/61242 PMC8284398 32628170

[36] Vang A , da Silva Gonçalves Bos D , Fernandez‐Nicolas A , et al. α7 nicotinic acetylcholine receptor mediates right ventricular fibrosis and diastolic dysfunction in pulmonary hypertension. JCI Insight. 2021;6: e142945. 33974567 10.1172/jci.insight.142945 PMC8262476

[37] Mendiola EA , Neelakantan S , Xiang Q , et al. An image‐driven micromechanical approach to characterize multiscale remodeling in infarcted myocardium. Acta Biomater. 2024;173:109‐122. 37925122 10.1016/j.actbio.2023.10.027 PMC10924194

[38] Holzapfel GA , Gasser TC , Ogden RW . A new constitutive framework for arterial wall mechanics and a comparative study of material models. J Elast Phys Sci Solids. 2000;61:1‐48.

[39] Dong H , Sun W . A novel hyperelastic model for biological tissues with planar distributed fibers and a second kind of poisson effect. J Mech Phys Solids. 2021;151:104‐377.

[40] Kailash KA , Hawes JZ , Cocciolone AJ , Bersi MR , Mecham RP , Wagenseil JE . Constitutive modeling of mouse arteries suggests changes in directional coupling and extracellular matrix remodeling that depend on artery type, age, sex, and elastin amounts. J Biomech Eng. 2024;146:060901. 10.1115/1.4063272 PMC12410933 37646627

[41] Caro CG , McDonald DA . The relation of pulsatile pressure and flow in the pulmonary vascular bed. J Physiol. 1961;157:426‐453. 13690903 10.1113/jphysiol.1961.sp006734 PMC1359986

[42] O'Rourke MF . Vascular impedance in studies of arterial and cardiac function. Physiol Rev. 1982;62:570‐623. 6461866 10.1152/physrev.1982.62.2.570

[43] Wang Z , Chesler NC . Pulmonary vascular wall stiffness: an important contributor to the increased right ventricular afterload with pulmonary hypertension. Pulm Circ. 2011;1:212‐223. 22034607 10.4103/2045-8932.83453 PMC3198648

[44] Althouse AD , Below JE , Claggett BL , et al. Recommendations for statistical reporting in cardiovascular medicine: a special report from the american heart association. Circulation. 2021;144:e70‐e91. 34032474 10.1161/CIRCULATIONAHA.121.055393 PMC12850682

[45] Hunter KS , Lee PF , Lanning CJ , et al. Pulmonary vascular input impedance is a combined measure of pulmonary vascular resistance and stiffness and predicts clinical outcomes better than pulmonary vascular resistance alone in pediatric patients with pulmonary hypertension. Am Heart J. 2008;155:166‐174. 18082509 10.1016/j.ahj.2007.08.014 PMC3139982

[46] Thenappan T , Prins KW , Pritzker MR , Scandurra J , Volmers K , Weir EK . The critical role of pulmonary arterial compliance in pulmonary hypertension. Ann Am Thorac Soc. 2016;13:276‐284. 26848601 10.1513/AnnalsATS.201509-599FR PMC5461956

[47] Hungerford SL , Kearney K , Song N , et al. Prognostic role of pulmonary impedance estimation to predict right ventricular dysfunction in pulmonary hypertension. ESC Heart Fail. 2023;10:1811‐1821. 36896830 10.1002/ehf2.14180 PMC10192280

[48] Qureshi MU , Colebank MJ , Schreier DA , et al. Characteristic impedance: frequency or time domain approach? Physiol Meas. 2018;39:014004. 29176040 10.1088/1361-6579/aa9d60 PMC5828940

[49] Rich S et al. Primary pulmonary hypertension: a national prospective study. Ann Int Med. 1987;107:216‐223. 3605900 10.7326/0003-4819-107-2-216

[50] Matura LA , McDonough A , Carroll DL . Cluster analysis of symptoms in pulmonary arterial hypertension: a pilot study. Eur J Cardiovasc Nurs. 2012;11:51‐61. 22357779 10.1177/1474515111429649

[51] Neelakantan S , Manning EP , Zhang P , Choudhary G , Avazmohammadi R . Abstract 14,407: right ventricular myocardial stiffening is associated with pulmonary arterial stiffening in pulmonary hypertension. Circulation. 2023;148:A14407.

[52] Mehdi RRR et al. Contractile adaptation of the right ventricular myocardium in pulmonary hypertension. Circ Res. 2023;133:AP2008.

[53] Schäfer M et al. Pulmonary arterial stiffness: toward a new paradigm in pulmonary arterial hypertension pathophysiology and assessment. Curr Hypertens Rep. 2016;18:4. 26733189 10.1007/s11906-015-0609-2

[54] Gupta A et al. Novel noninvasive assessment of pulmonary arterial stiffness using velocity transfer function. J Am Heart Assoc. 2018;7:e009459. 30371198 10.1161/JAHA.118.009459 PMC6222968

