# Supplementary material for: Dissecting contributions of pulmonary arterial remodeling to right ventricular afterload in pulmonary hypertension
Source: Bioeng Transl Med. 2025 Jun 26;10(4):e70035. doi: 10.1002/btm2.70035 (PMC12284438; doi:10.1002/btm2.70035)
Supplement: Supplementary file 1 — Data S1: Supporting Information. [file BTM2-10-e70035-s001.pdf]

Supplementary Material

S.1 Non-linear and anisotropic constitutive model for the pulmonary artery

The isotropic and anisotropic strain energy functions are given by:

$$\overline{\Psi}_{iso} = \frac{c}{2}(\bar{I}_1 - 3), \tag{1}$$

$$\overline{\Psi}_{aniso} = \frac{k_1}{2k_2}\{\exp[k_2(\bar{I}_4 - 1)^2] + \exp[k_2(\bar{I}_6 - 1)^2] - 2\}, \tag{2}$$

where c, k_1 and k_2 are material parameters. c is associated with the isotropic (ECM) response, while k_1 and k_2 are associated with the fiber (anisotropic) response. The Cauchy stress is given by

$$\overline{\sigma} = c\ dev(\overline{\mathbf{b}}) + 2\overline{\psi}_4\ dev(\mathbf{a}_1 \otimes \mathbf{a}_1) + 2\overline{\psi}_6\ dev(\mathbf{a}_2 \otimes \mathbf{a}_2), \tag{3}$$

$$\overline{\mathbf{b}} = \overline{\mathbf{F}}\overline{\mathbf{F}}^{\mathbf{T}}, \tag{4}$$

$$dev(\bullet) = (\bullet) - \frac{1}{3}[(\bullet) : \mathbf{I}]\mathbf{I}, \tag{5}$$

where $\overline{\psi}_4 = \partial\overline{\Psi}_{aniso}/\partial\bar{I}_4$ and $\overline{\psi}_6 = \partial\overline{\Psi}_{aniso}/\partial\bar{I}_6$ are the derivatives of the anisotropic strain energy function with respect to the invariants $\bar{I}_4$ and $\bar{I}_6$. $\overline{\mathbf{b}}$ is the left Cauchy-green tensor. The derivatives were computed to be

$$\frac{\partial\overline{\Psi}_{aniso}}{\partial\bar{I}_4} = k_1(\bar{I}_4 - 1)\exp[k_2(\bar{I}_4 - 1)^2], \tag{6}$$

$$\frac{\partial\overline{\Psi}_{aniso}}{\partial\bar{I}_6} = k_1(\bar{I}_6 - 1)\exp[k_2(\bar{I}_6 - 1)^2]. \tag{7}$$

For an ideal case, assume the following isochoric deformation vector with no shear components

$$\overline{\mathbf{F}} = \begin{bmatrix} \lambda_r & 0 & 0 \\ 0 & \lambda_\theta & 0 \\ 0 & 0 & \lambda_z \end{bmatrix}, \qquad \text{where} \quad \lambda_r\lambda_\theta\lambda_z = 1, \tag{8}$$

$$\overline{\mathbf{b}} \ = \ \overline{\mathbf{C}} = \begin{bmatrix} \lambda_r^2 & 0 & 0 \\ 0 & \lambda_\theta^2 & 0 \\ 0 & 0 & \lambda_z^2 \end{bmatrix}, \tag{9}$$

$$dev(\overline{\mathbf{b}}) = \begin{bmatrix} \frac{2}{3}\lambda_r^2 - \frac{1}{3}\left(\lambda_\theta^2 + \lambda_z^2\right) & 0 & 0 \\ 0 & \frac{2}{3}\lambda_\theta^2 - \frac{1}{3}\left(\lambda_r^2 + \lambda_z^2\right) & 0 \\ 0 & 0 & \frac{2}{3}\lambda_z^2 - \frac{1}{3}\left(\lambda_r^2 + \lambda_\theta^2\right) \end{bmatrix}. \tag{10}$$

Hence, the isotropic stress is given by

$$\overline{\sigma}_{iso} = c \begin{bmatrix} \frac{2}{3}\lambda_r^2 - \frac{1}{3}\left(\lambda_\theta^2 + \lambda_z^2\right) & 0 & 0 \\ 0 & \frac{2}{3}\lambda_\theta^2 - \frac{1}{3}\left(\lambda_r^2 + \lambda_z^2\right) & 0 \\ 0 & 0 & \frac{2}{3}\lambda_z^2 - \frac{1}{3}\left(\lambda_r^2 + \lambda_\theta^2\right) \end{bmatrix}. \tag{11}$$

Considering the effects of the fibers, $\mathbf{a}_1 = \overline{\mathbf{F}} a_{01}$, where a_{01} and a_{02} are given by the fiber angle β.

$$a_{01} = \begin{bmatrix} 0 \\ \cos\beta \\ \sin\beta \end{bmatrix}, \qquad a_{02} = \begin{bmatrix} 0 \\ \cos\beta \\ -\sin\beta \end{bmatrix}, \tag{12}$$

$$\mathbf{a}_1 = \begin{bmatrix} \lambda_r & 0 & 0 \\ 0 & \lambda_\theta & 0 \\ 0 & 0 & \lambda_z \end{bmatrix} \begin{bmatrix} 0 \\ \cos\beta \\ \sin\beta \end{bmatrix}, \qquad \mathbf{a}_2 = \begin{bmatrix} \lambda_r & 0 & 0 \\ 0 & \lambda_\theta & 0 \\ 0 & 0 & \lambda_z \end{bmatrix} \begin{bmatrix} 0 \\ \cos\beta \\ -\sin\beta \end{bmatrix}, \tag{13}$$

$$\mathbf{a}_1 = \begin{bmatrix} 0 \\ \lambda_\theta\cos\beta \\ \lambda_z\sin\beta \end{bmatrix}, \qquad \mathbf{a}_2 = \begin{bmatrix} 0 \\ \lambda_\theta\cos\beta \\ -\lambda_z\sin\beta \end{bmatrix}. \tag{14}$$

Now converting $\mathbf{a}_1$ and $\mathbf{a}_2$ into matrices and determining the deviatoric part

$$\mathbf{a}_1 \otimes \mathbf{a}_1 = \begin{bmatrix} 0 & 0 & 0 \\ 0 & (\lambda_\theta\cos\beta)^2 & \lambda_\theta\lambda_z\cos\beta\sin\beta \\ 0 & \lambda_\theta\lambda_z\cos\beta\sin\beta & (\lambda_z\sin\beta)^2 \end{bmatrix}, \tag{15}$$

$$dev(\mathbf{a}_1 \otimes \mathbf{a}_1) = \begin{bmatrix} -\frac{1}{3}\left((\lambda_\theta\cos\beta)^2 + (\lambda_z\sin\beta)^2\right) & 0 & 0 \\ 0 & \frac{2}{3}(\lambda_\theta\cos\beta)^2 - \frac{1}{3}(\lambda_z\sin\beta)^2 & \lambda_\theta\lambda_z\cos\beta\sin\beta \\ 0 & \lambda_\theta\lambda_z\cos\beta\sin\beta & \frac{2}{3}(\lambda_z\sin\beta)^2 - \frac{1}{3}(\lambda_\theta\cos\beta)^2 \end{bmatrix}, \tag{16}$$

$$\mathbf{a}_2 \otimes \mathbf{a}_2 = \begin{bmatrix} 0 & 0 & 0 \\ 0 & (\lambda_\theta\cos\beta)^2 & -\lambda_\theta\lambda_z\cos\beta\sin\beta \\ 0 & -\lambda_\theta\lambda_z\cos\beta\sin\beta & (\lambda_z\sin\beta)^2 \end{bmatrix}, \tag{17}$$

$$dev(\mathbf{a}_2 \otimes \mathbf{a}_2) = \begin{bmatrix} -\frac{1}{3}\left((\lambda_\theta\cos\beta)^2 + (\lambda_z\sin\beta)^2\right) & 0 & 0 \\ 0 & \frac{2}{3}(\lambda_\theta\cos\beta)^2 - \frac{1}{3}(\lambda_z\sin\beta)^2 & -\lambda_\theta\lambda_z\cos\beta\sin\beta \\ 0 & -\lambda_\theta\lambda_z\cos\beta\sin\beta & \frac{2}{3}(\lambda_z\sin\beta)^2 - \frac{1}{3}(\lambda_\theta\cos\beta)^2 \end{bmatrix}. \tag{18}$$

Now computing the stress $\sigma_{\theta\theta}$

$$\begin{aligned}\overline{\sigma}_{rr} =\ & c\left[\frac{2}{3}\lambda_r^2 - \frac{1}{3}\left(\lambda_\theta^2+\lambda_z^2\right)\right] - \frac{2k_1(\bar{I}_4-1)}{3}\exp[k_2(\bar{I}_4-1)^2]\left[(\lambda_\theta\cos\beta)^2+(\lambda_z\sin\beta)^2\right] \\ & -\frac{2k_1(\bar{I}_6-1)}{3}\exp[k_2(\bar{I}_6-1)^2]\left[(\lambda_\theta\cos\beta)^2+(\lambda_z\sin\beta)^2\right],\end{aligned} \tag{19}$$

$$\begin{aligned}\overline{\sigma}_{\theta\theta} =\ & c\left[\frac{2}{3}\lambda_\theta^2 - \frac{1}{3}\left(\lambda_r^2+\lambda_z^2\right)\right] + \frac{2k_1(\bar{I}_4-1)}{3}\exp[k_2(\bar{I}_4-1)^2]\left[2\,(\lambda_\theta\cos\beta)^2-(\lambda_z\sin\beta)^2\right] \\ & +\frac{2k_1(\bar{I}_6-1)}{3}\exp[k_2(\bar{I}_6-1)^2]\left[2\,(\lambda_\theta\cos\beta)^2-(\lambda_z\sin\beta)^2\right],\end{aligned} \tag{20}$$

$$\begin{aligned}\overline{\sigma}_{zz} =\ & c\left[\frac{2}{3}\lambda_z^2 - \frac{1}{3}\left(\lambda_r^2+\lambda_\theta^2\right)\right] + \frac{2k_1(\bar{I}_4-1)}{3}\exp[k_2(\bar{I}_4-1)^2]\left[2\,(\lambda_z\sin\beta)^2-(\lambda_\theta\cos\beta)^2\right] \\ & +\frac{2k_1(\bar{I}_6-1)}{3}\exp[k_2(\bar{I}_6-1)^2]\left[2\,(\lambda_z\sin\beta)^2-(\lambda_\theta\cos\beta)^2\right].\end{aligned} \tag{21}$$

Now computing the fiber-related invariants

$$\bar{I}_4 = \overline{\mathbf{C}} : (a_{01}\otimes a_{01}), \quad \text{and} \quad \bar{I}_6 = \overline{\mathbf{C}} : (a_{02}\otimes a_{02}), \tag{22}$$

$$\Longrightarrow \bar{I}_4 = \lambda_\theta^2\cos^2\beta+\lambda_z^2\sin^2\beta, \quad \text{and} \quad \bar{I}_6 = \lambda_\theta^2\cos^2\beta+\lambda_z^2\sin^2\beta. \tag{23}$$

The final stress values are

$$\begin{aligned}\overline{\sigma}_{rr} =\ & c\left[\frac{2}{3}\lambda_r^2 - \frac{1}{3}\left(\lambda_\theta^2+\lambda_z^2\right)\right] \\ & -\frac{4k_1}{3}\exp[k_2\left(\left[\lambda_\theta^2\cos^2\beta+\lambda_z^2\sin^2\beta\right]-1\right)^2]\left[(\lambda_\theta\cos\beta)^2+(\lambda_z\sin\beta)^2\right]\left[\lambda_\theta^2\cos^2\beta+\lambda_z^2\sin^2\beta-1\right],\end{aligned} \tag{24}$$

$$\begin{aligned}\overline{\sigma}_{\theta\theta} =\ & c\left[\frac{2}{3}\lambda_\theta^2 - \frac{1}{3}\left(\lambda_r^2+\lambda_z^2\right)\right] \\ & +\frac{4k_1}{3}\exp[k_2\left(\left[\lambda_\theta^2\cos^2\beta+\lambda_z^2\sin^2\beta\right]-1\right)^2]\left[2\,(\lambda_\theta\cos\beta)^2-(\lambda_z\sin\beta)^2\right]\left[\lambda_\theta^2\cos^2\beta+\lambda_z^2\sin^2\beta-1\right],\end{aligned} \tag{25}$$

$$\begin{aligned}\overline{\sigma}_{zz} =\ & c\left[\frac{2}{3}\lambda_z^2 - \frac{1}{3}\left(\lambda_r^2+\lambda_\theta^2\right)\right] \\ & +\frac{4k_1}{3}\exp[k_2\left(\left[\lambda_\theta^2\cos^2\beta+\lambda_z^2\sin^2\beta\right]-1\right)^2]\left[2\,(\lambda_z\sin\beta)^2-(\lambda_\theta\cos\beta)^2\right]\left[\lambda_\theta^2\cos^2\beta+\lambda_z^2\sin^2\beta-1\right].\end{aligned} \tag{26}$$

We know that $\sigma = \sigma_{vol}+\overline{\sigma}$, where $\sigma_{vol} = p\mathbf{I}$ is the hydrostatic stress and p is the hydrostatic pressure.

S.2 Fluid pressure during inflation of vessel

For pure inflation of a cylinder, the variables in the deformed configuration are given by $r = r(R)$, $\theta = \Theta$, and $z = Z$. Here $R \in [R_i, R_o]$, $\Theta \in [0, 2\pi]$ and let $R_o - R_i = h$ be the thickness. Hence, the deformation gradient and Cauchy green tensors are given by

$$\overline{\mathbf{F}} = \begin{bmatrix} \frac{R}{r} & 0 & 0 \\ 0 & \frac{r}{R} & 0 \\ 0 & 0 & 1 \end{bmatrix}, \tag{27}$$

$$\overline{\mathbf{b}} = \begin{bmatrix} \left(\frac{R}{r}\right)^2 & 0 & 0 \\ 0 & \left(\frac{r}{R}\right)^2 & 0 \\ 0 & 0 & 1 \end{bmatrix}. \tag{28}$$

We can compute hydrostatic pressure using the equilibrium equation $\nabla\sigma = 0$. In cylindrical coordinates

$$\frac{\partial \sigma_{rr}}{\partial r} + \frac{\sigma_{rr} - \sigma_{\theta\theta}}{r} = 0. \tag{29}$$

We use this to get the value of internal pressure as

$$P_i = \int_{r_i}^{r_o} \frac{\sigma_{\theta\theta} - \sigma_{rr}}{r}\, dr. \tag{30}$$

We can substitute $\sigma_{rr} - \sigma_{\theta\theta} = \overline{\sigma}_{rr} - \overline{\sigma}_{\theta\theta}$, because $\sigma_{rr} = \overline{\sigma}_{rr} + p$, and $\sigma_{\theta\theta} = \overline{\sigma}_{\theta\theta} + p$, where p is the hydrostatic pressure. Hence

$$P_i = \int_{r_i}^{r_o} \frac{\overline{\sigma}_{\theta\theta} - \overline{\sigma}_{rr}}{r}\, dr, \tag{31}$$

Computing the RHS

$$\begin{aligned} \overline{\sigma}_{\theta\theta} - \overline{\sigma}_{rr} = {} & c\left[\lambda_\theta^2 - \lambda_r^2\right] \\ & + \frac{4k_1}{3}\exp[k_2\left(\left[\lambda_\theta^2\cos^2\beta + \lambda_z^2\sin^2\beta\right] - 1\right)^2]\left[3\left(\lambda_\theta\cos\beta\right)^2\right]\left[\lambda_\theta^2\cos^2\beta + \lambda_z^2\sin^2\beta - 1\right]. \end{aligned} \tag{32}$$

Substituting the values for λ_r, λ_θ, λ_z, we get

$$\begin{aligned} \overline{\sigma}_{\theta\theta} - \overline{\sigma}_{rr} = {} & c\left[\left(\frac{r}{R}\right)^2 - \left(\frac{R}{r}\right)^2\right] \\ & + \frac{4k_1}{3}\exp\left[k_2\left(\left[\left(\frac{r}{R}\right)^2\cos^2\beta + \sin^2\beta\right] - 1\right)^2\right]\left[3\left(\left(\frac{r}{R}\right)\cos\beta\right)^2\right]\left[\left(\frac{r}{R}\right)^2\cos^2\beta + \sin^2\beta - 1\right]. \end{aligned} \tag{33}$$

We can compute P_i by integrating Eq. S31 over the thickness of the vessel.

S.3 Distribution of vessel length and radius

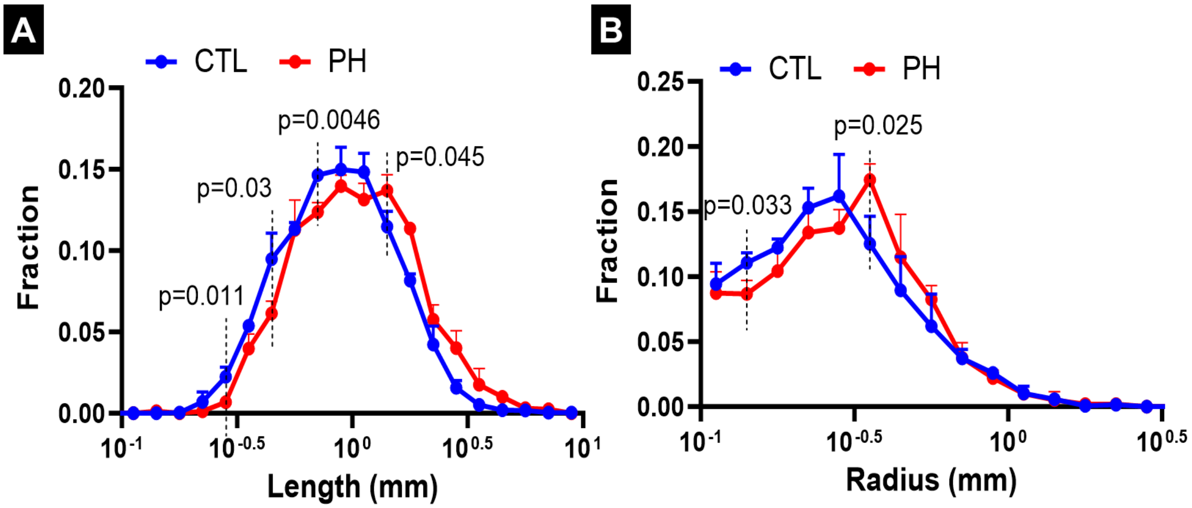

Figure S1. Distribution of (**A**) length and (**B**) radius of the vessels in the pulmonary vasculature. Analysis was performed using ex vivo μCT images from fixed lungs. CTL: n=3, PH: n=3. Statistical significance is presented at representative points, estimated through Student's t-test.

S.4 Ex-vivo analysis

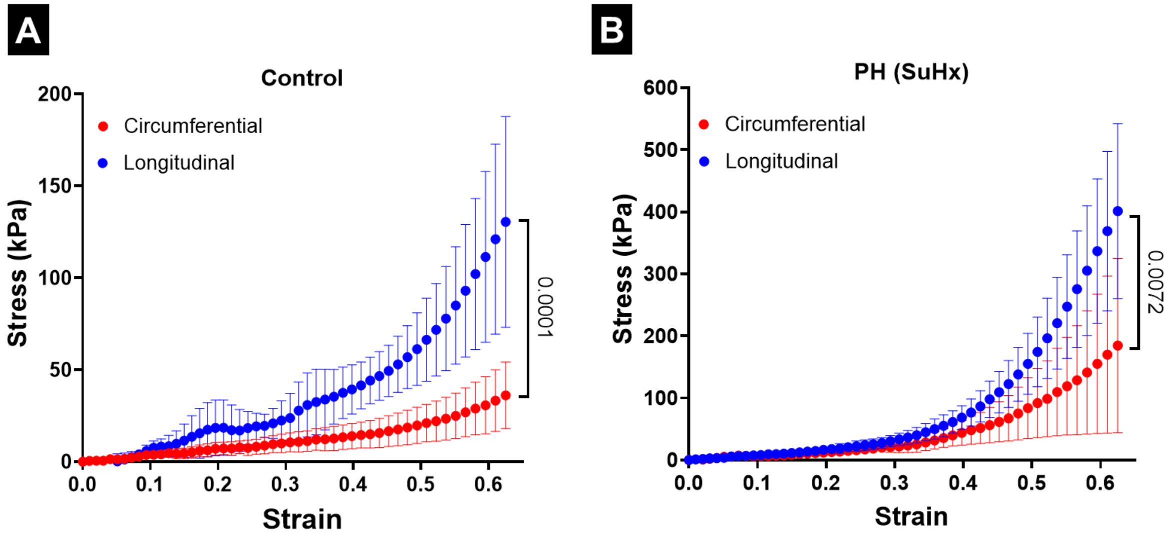

Figure S2. Mean stress-strain relation of MPA tissue specimens from (**A**) control rats and (**B**) PH rats acquired from uniaxial testing. Stress reported here is the 2nd-Piola Kirchhoff stress, and strain reported here is the Green-Lagrange strain. CTL: n=8, PH: n=8.

Table S1. Material parameters (mean ± SD) obtained by fitting the constitutive model to individual stress strain curves. n=8 control, n=8 PH.

Material Parameter	CTL	PH
c (kPa)	9.37±1.77	19.38±3.20
k_1 (kPa)	102.5±59.52	287.5±228.21
k_2	4.63±1.48	17±8.3
θ (°)	48.44±1.08	46.85±1.45

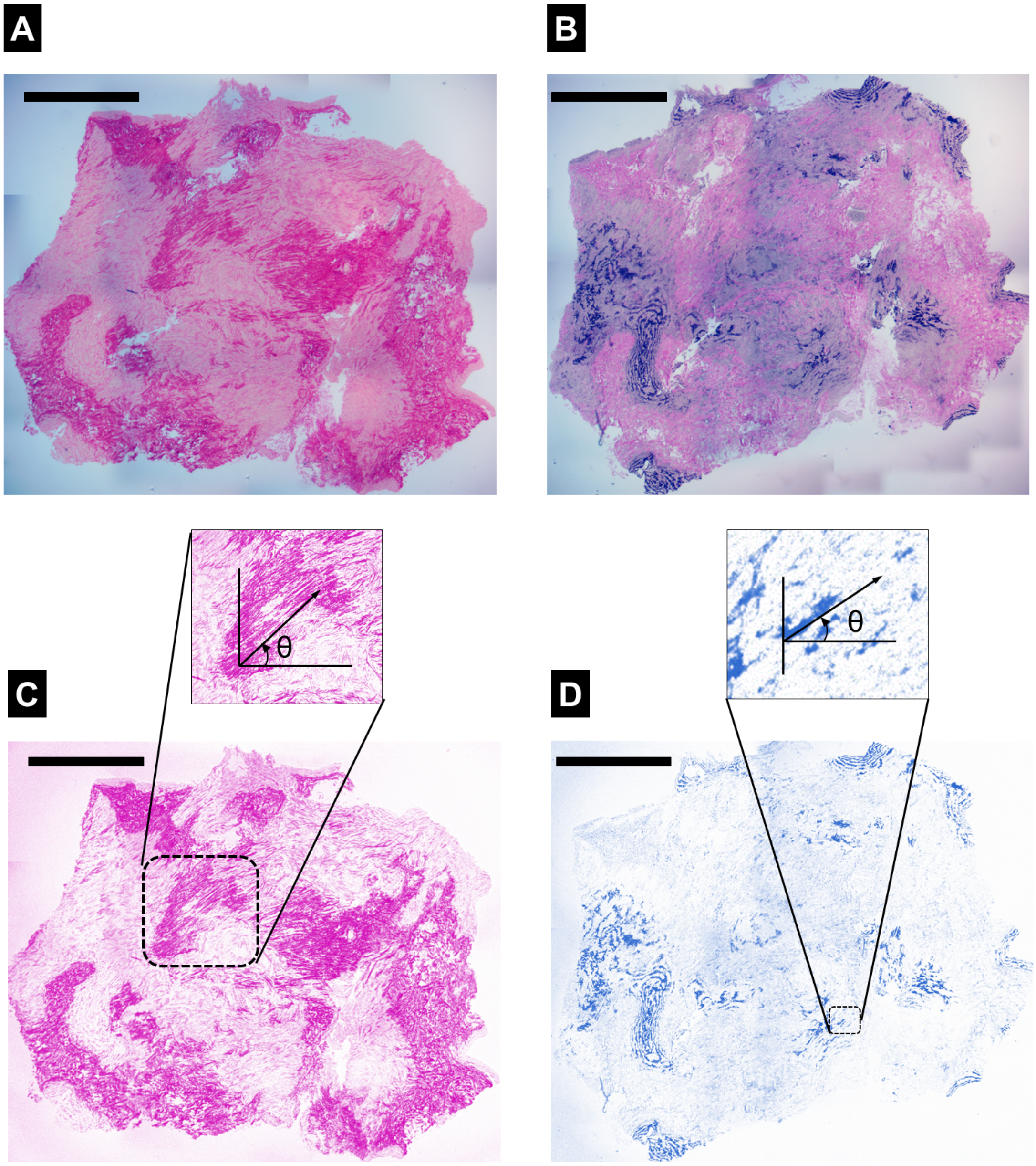

Figure S3. Representative histological images stained with (**A**) Picrosirius Red (PSR) and (**B**) Verhoeff-Van Gieson (VVG). (**C**) Collagen content isolated through image analysis. (**D**) Elastin content isolated through image analysis. The inset figures represent the fiber orientation of a small region of interest. Scale bar indicates 0.5 mm.

S.5 Effect of remodeling parameters on pressure waveform

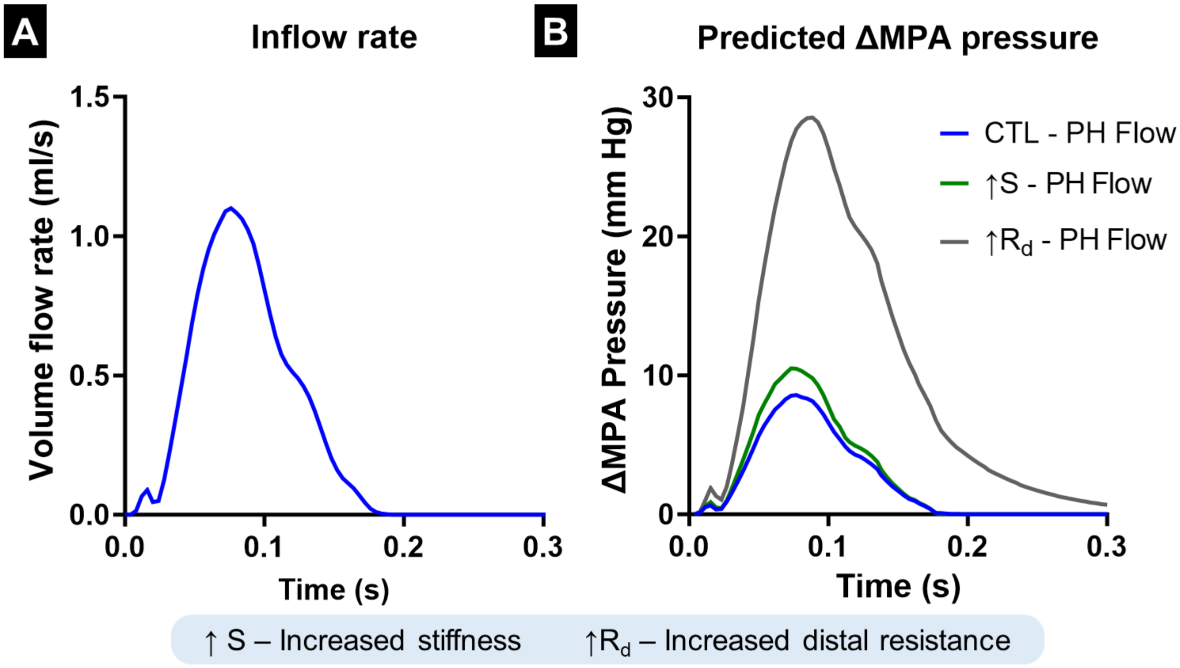

Figure S4. Effect of individual vascular remodeling events on the pressure waveform. (**A**) Volume flow rate at the inlet, (**B**) Pressure waveform for increased vessel stiffness and distal resistance. (**C**) Volume flow rate at the inlet with an increased period of zero flow after the pulse, (**B**) Pressure waveform increased distal resistance to study the relaxation behavior.

S.6 Variation of PA impedance with frequency

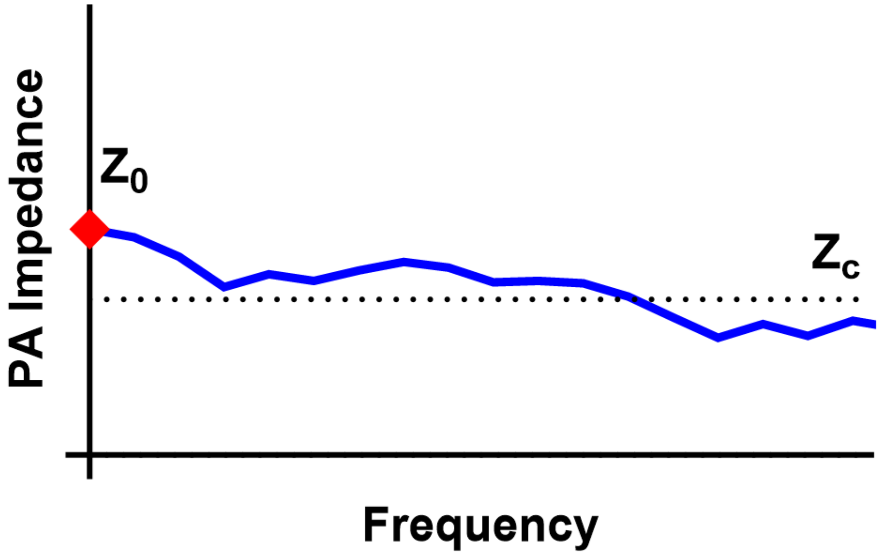

Figure S5. Schematic describing the variation of impedance with frequency and the estimation of 0Hz- and characteristic impedance.

S.7 Verification against 3D FSI

For verfication, we have compared the 1D FSI framework presented in this study against a 3D FSI framework. Here we simulated a constant fluid flow rate through a Y-branch, where the length and radius of each vessel are 50mm and 5mm respectively. The thickness is set to be 20% of the radius (1mm). The flow rate is set to 30ml/s. For the vessel properties, the stiffness of fiber components was set to 0, and the shear modulus (c) was set to 60kPa. To perform the equivalent simulation in 3D, we performed the simulation in ANSYS workbench, coupling the transient structural and Fluent modules. Here, the displacement of the inlet and outlet faces was constrained such that only radial and circumferential displacement was allowed. A single additional point on the inlet was completely fixed to ensure stable simulations.

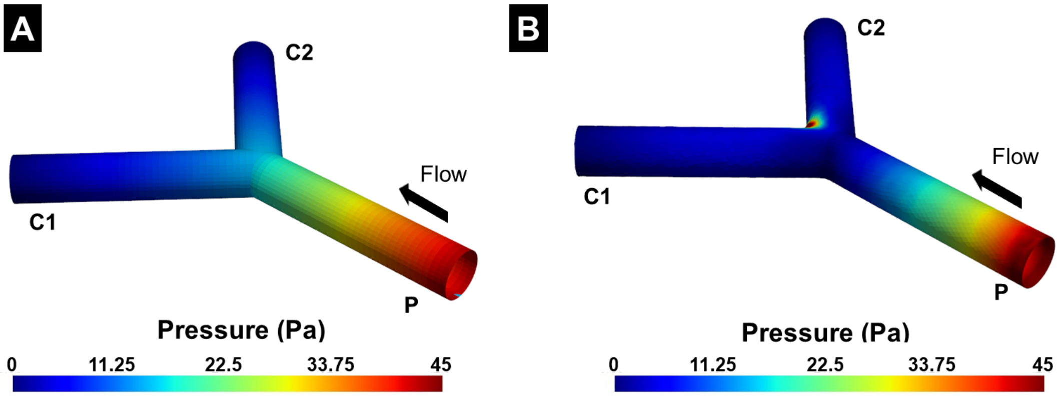

Figure S6. (A) The pressure predicted by the 1D simulation mapped onto a representative geometry, and (B) Pressure on the wall of the fluid domain. **P**: Parent vessel; **C1**, **C2**: child vessels.

The results indicated good agreement between the 1D and 3D simulations. The pressure at the inlet in the 1D simulation was 42.8 Pa. Correspondingly, the pressure on the fluid domain wall from the 3D simulation was 44.7Pa. The difference between the predicted values is likely due to the idealized bifurcation assumed by the 1D FSI framework.

S.8 Effect of non-linear material model

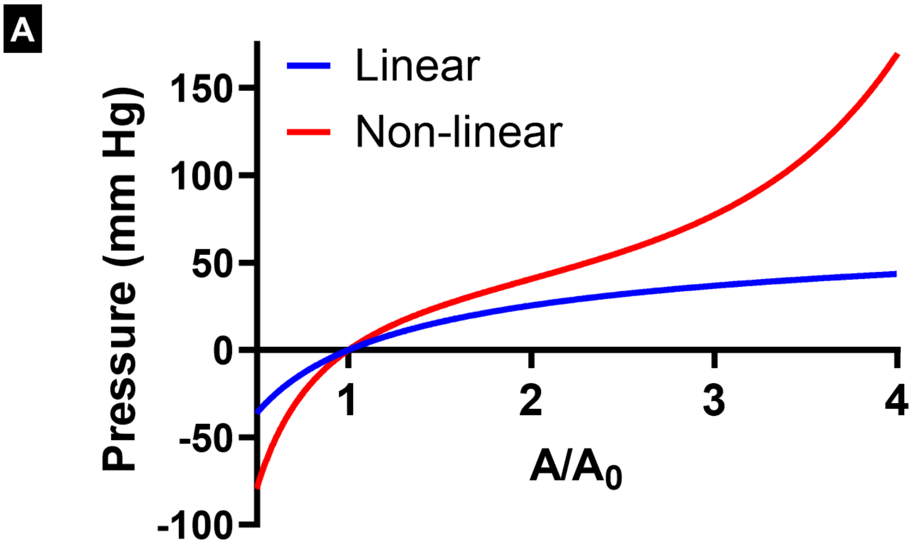

Figure S7. Variation of pressure with lumen area. Lumen area was normalized by the original lumen area
